# Carbon Fate and Flux in *Prochlorococcus* under Nitrogen Limitation

**DOI:** 10.1128/mSystems.00254-18

**Published:** 2019-02-26

**Authors:** Martin J. Szul, Stephen P. Dearth, Shawn R. Campagna, Erik R. Zinser

**Affiliations:** a Department of Microbiology, University of Tennessee, Knoxville, Tennessee, USA; b Department of Microbiology and Immunology, College of Graduate Studies, Midwestern University, Downers Grove, Illinois, USA; c Department of Chemistry, University of Tennessee, Knoxville, Tennessee, USA; d Biological and Small Molecule Mass Spectrometry Core, University of Tennessee, Knoxville, Tennessee, USA; City of Knowledge

**Keywords:** *Prochlorococcus*, carbon, cyanobacteria, metabolomics, nitrogen

## Abstract

Primary production by *Prochlorococcus*, the smallest known free-living photosynthetic organism in terms of both physical and genomic size, is thought to have a significant role in global carbon cycles. Despite its small size and low growth rate, *Prochlorococcus* numerically dominates the phytoplankton community in the nutrient-poor oligotrophic ocean, the largest biome of the Earth’s surface. How nutrient limitation, and nitrogen limitation in particular, affects the fate and flux of carbon fixed by *Prochlorococcus* is currently unknown. To address this gap in knowledge, we compared the bulk rates of photosynthesis and organic carbon release, the concentrations of intracellular metabolites, and the rates of assimilated carbon into the metabolite pools between replete and N-limited chemostat cultures. Total photosynthesis of our N-limited cultures was less than half of those observed in replete cultures, and nitrogen limitation also appears to cause a larger proportion of total fixed carbon to be released to the environment. Our data suggest this occurs in concert with the maintenance of large slow-moving pools of metabolites, including nitrogen-rich molecules such as glutamate. Additionally, we report field data suggesting metabolisms of *Prochlorococcus* are comparable to results we observe in our laboratory studies. Accounting for these observations, potential metabolic mechanisms utilized by *Prochlorococcus* are discussed as we build upon our understanding of nutrient-limited photosynthesis and carbon metabolism.

**IMPORTANCE** Photosynthetic microbes are the predominant sources of organic carbon in the sunlit regions of the ocean. During photosynthesis, nitrogen and carbon metabolism are coordinated to synthesize nitrogen-containing organics such as amino acids and nucleic acids. In large regions of the ocean, nitrogen is thought to limit the growth of phytoplankton. The impact of nitrogen limitation on the synthesis of organic carbon is not well understood, especially for the most abundant photosynthetic organism in the nitrogen-limited regions of the ocean, *Prochlorococcus*. This study compares the carbon metabolism of nitrogen-replete and nitrogen-limited *Prochlorococcus* spp. to determine how nitrogen availability influences inorganic carbon assimilation into an organic form. Metabolomics and physiological data revealed that cells under nitrogen limitation have reduced metabolic flux and total carbon fixation rates while maintaining elevated metabolite pool levels and releasing a larger proportion of total fixed carbon to the environment.

## Abstract (precis)

Photosynthetic microbes are the predominant sources of organic carbon in the sunlit regions of the ocean. During photosynthesis, nitrogen and carbon metabolism are coordinated to synthesize nitrogen-containing organics such as amino acids and nucleic acids. In large regions of the ocean, nitrogen is thought to limit the growth of phytoplankton. The impact of nitrogen limitation on the synthesis of organic carbon is not well understood, especially for the most abundant photosynthetic organism in the nitrogen-limited regions of the ocean, *Prochlorococcus*. This study compares the carbon metabolism of nitrogen-replete and nitrogen-limited *Prochlorococcus* spp. to determine how nitrogen availability influences inorganic carbon assimilation into an organic form. Metabolomics and physiological data revealed that cells under nitrogen limitation have reduced metabolic flux and total carbon fixation rates while maintaining elevated metabolite pool levels and releasing a larger proportion of total fixed carbon to the environment.

## INTRODUCTION

As the numerically dominant phytoplankton in the world’s tropical and subtropical oligotrophic oceans (1, 2), *Prochlorococcus* spp. are suspected to function as key nodes in community food webs and drive productivity in one of Earth’s largest biomes. The sunlit blue waters where these phytoplankton thrive are characterized by persistent and regionally specific nutrient limitations (3). Factors such as upwelling, aeolian transport, and physical mixing of water masses can generate nutrient limitation for phosphorus or iron in some regions and for nitrogen in other regions, such as the North Pacific (3–5). It has been proposed that both small genomes and cell sizes may improve both thrift and competition for scarce nutrients in these environments; consistent with this hypothesis, the most abundant bacterial members of these communities have minimal (streamlined) genomes and are among the smallest known free-living organisms (6). The impact that reduced genomes and cell sizes have on the utilization of nutrient resources toward biomass production is not well understood due to the paucity of empirical data on the orchestration of carbon and nitrogen metabolism in *Prochlorococcus* under nitrogen limitation.

In the canonical cyanobacterial system (7), nitrogen limitation leads to the pooling of 2-oxoglutarate (2OG), which in turn serves as the intracellular signal for nitrogen-limiting conditions. The signal upregulates nitrogen assimilation by modulating the activity of three cyanobacterial regulators, namely, NtcA, PipX, and P_II_ (7–10). Briefly, this occurs as nitrogen limitation restricts the NH_4_^+^ assimilatory reactions catalyzed by glutamine synthetase (GS) and glutamine-oxoglutarate amidotransferase (GOGAT), leading to increased intracellular concentrations of 2OG. NtcA, the global nitrogen regulator, becomes activated as this protein binds with 2OG and PipX. This complex is then involved in upregulation of both itself (*ntcA*) and nitrogen assimilatory mechanisms (e.g., nitrogen acquisition, carbon fixation, and GS). In addition to the activation of NtcA, 2OG can bind P_II_, and this complex sequesters the P_II_ signal transduction protein from its capability to signal nitrogen availability. When nitrogen is abundant, high activity of GS-GOGAT draws down pools of 2OG, reducing the association of 2OG and P_II_. Free P_II_ promotes the synthesis of nitrogen-rich arginine by binding and promoting the key biosynthetic enzyme *N*-acetyl-glutamate-kinase (8, 11). Arginine and aspartate formed under N replete conditions, along with their roles in protein biosynthesis, can serve as precursors for nitrogen storage metabolites (e.g., polyamines).

Previous investigations into the regulation of nitrogen acquisition genes provide evidence that *Prochlorococcus* transcribes the three known regulatory proteins, P_II_, PipX, and NtcA, although these regulators have not been observed to function as observed in other systems. For example, recent studies on related strains of *Prochlorococcus* suggest that the metabolite 2OG can interact with NtcA, the global nitrogen regulator, enhancing complex binding to one of its target genes, *glnA*, albeit the strength of binding is less than has been reported in other cyanobacteria (12). Lindell et al. provided data suggesting, as observed in other cyanobacterial systems, that the transcription of *ntcA* is upregulated under nitrogen stress, but they also showed that the transcriptional abundance of the ammonia transporter gene *amt1* is not correlated with *ntcA* and appears to be constitutively expressed (13). Furthermore, proteomic studies on N stressed cultures of *Prochlorococcus* sp. strain SS120 suggest NtcA is upregulated while levels of both PipX and P_II_ are suppressed (14); contrary to what has been observed in other cyanobacteria, Palinska et al. have shown that P_II_ in *Prochlorococcus* does not undergo phosphorylation in response to the species of nitrogen available (15). Additional studies show contrasting results where nitrogen stress has been observed to induce GS transcription upregulation as observed in canonical systems (16), but both abundance and activity of GS do not appear to respond in kind (17). The transcription of genes involved in carbon acquisition and fixation suggest strain MED4 decreases both carbon fixation and glycogen synthesis during N stress (16). Although these carbon and nitrogen regulatory systems do not behave exactly as observed in other cyanobacterial systems, they are retained in the genomes of *Prochlorococcus* and thus are suspected to have important roles in the balance of carbon and nitrogen metabolism.

Nitrogen status can impact the release of organic carbon from photosynthetic microbes. More than 50 years ago, Fogg et al. (18) first reported photosynthate release to the environment as exudates by both healthy and stressed phytoplankton, and the amount of released exudate is directly correlated with stress (see reference 19 for a recent review). The leakage of fixed carbon has led to hypotheses that the loss of photosynthate may be attributed to an unavoidable passive diffusion of metabolites to the environment akin to a “property tax” (20), but this proposed mechanism fails to describe the effect of stress on exudate release. More recently, the paradox of organic carbon excretion under nutrient limitation was proposed to function as a form of “carbon exhaust.” In this model increased metabolic rates improve nutrient affinity, which in turn decreases the concentration of limiting nutrients necessary to support growth (21). An alternate hypothesis suggests that when energy input surpasses nutrient limited energy demand, cells may also fix inorganic carbon as a means to dissipate surplus energy while photosynthetic machinery is maintained. In this model, growth can be maximized when nutrients become available by reducing lag phase (22). Irrespective of the mechanism, metabolic by-products released to the environment as exudates fuel heterotrophic growth which in turn can support *Prochlorococcus* growth (21, 23).

Our ignorance regarding both the intracellular and the community-wide effects of carbon metabolism in *Prochlorococcus* under nitrogen limitation drove this research to develop a greater understanding of the metabolic mechanisms employed. To generate a deeper understanding of photosynthesis under nitrogen limitation, recently isolated axenic cultures of *Prochlorococcus* sp. strain VOL29 (eMED4/HL-I ecotype [24]) were grown in nitrogen-limited continuous chemostats and nitrogen-replete batch cultures. We measured photosynthetic rates and intracellular metabolite pool sizes and monitored stable isotopic carbon labeling for a subset of metabolites. These data provide evidence that nitrogen limitation reduces photosynthetic rates in *Prochlorococcus* while the concentrations of metabolic intermediates increase to form large pools with low turnover. Additionally, we report a larger proportion of carbon fixed by N-limited cultures is released as exudates than observed in replete cultures. These observations were compared against metabolomics data collected during a recent cruise in the geographic region (oligotrophic North Pacific) from which the isolate used in this study, *Prochlorococcus* VOL 29, was cultured (24). Data presented here describe metabolite-labeling patterns for *in vivo Prochlorococcus* populations to suggest their metabolisms follow similar trends to those observed in laboratory cultures.

## RESULTS AND DISCUSSION

### Nitrogen deficiency limits primary production and enhances organic carbon release.

**FIG 1:**
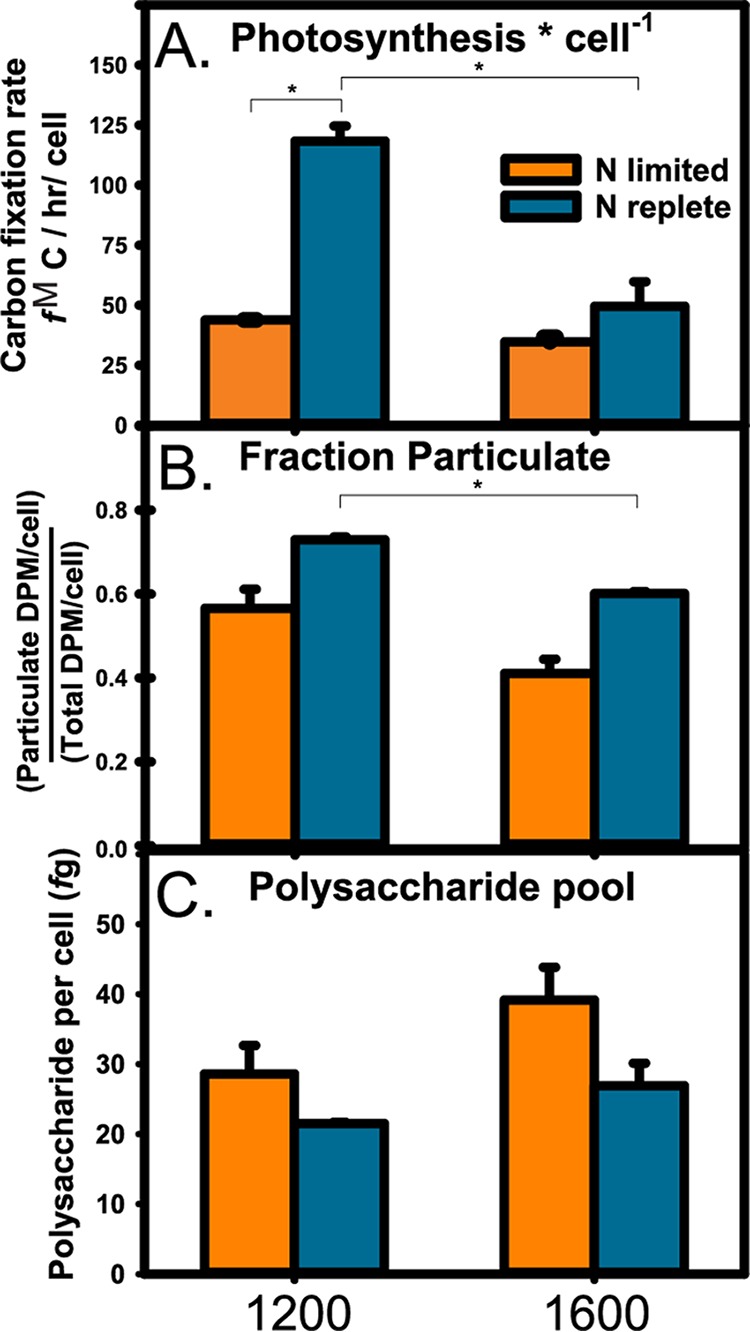
Photosynthesis and cellular carbon and energy stores in *Prochlorococcus* strain VOL29. Comparisons of the temporal effects (at 12:00 and 16:00, left and right, respectively) and nitrogen availability (limiting and replete; orange and blue, respectively) on total carbon fixation rates (A), the fraction of fixed carbon that is associated observed in the particulate (>0.2 μm) (B), and cellular carbon and energy stores determined through measurements of total polysaccharides (C). *, *P* < 0.05 by Welch’s *t* test.

*Prochlorococcus* VOL29 was grown in both ammonia-limited continuous culture and nutrient-replete batch culture to compare carbon metabolism under nitrogen-limited and -replete conditions. Cultures were grown in cycles of 14 h light/10 h dark and assayed principally at experimental noon and afternoon (12:00 and 16:00, respectively). In N-replete cultures, the rate of photosynthetic carbon fixation was higher at noon, dropping over 50% by 16:00 (Fig. 1A). A similar afternoon decline was observed in N-replete cultures of the related *Prochlorococcus* strain MED4 (25, 26). Carbon fixation is thought to slow during the afternoon as the cell shifts its photic energy utilization away from the Calvin cycle and toward the polymerization of macromolecules (e.g., DNA and glycogen) from the monomeric precursors generated earlier (25–27). Consistently, the polysaccharide (glycogen) pool in N-replete VOL29 was higher at 16:00 relative to that at 12:00 (*P* = 0.25) (Fig. 1C).

Growth under nitrogen limitation resulted in dramatic changes in carbon metabolism in VOL29. At noon, the rate of photosynthetic carbon fixation was less than 60% of the rate observed in N-replete cultures (Fig. 1A). Despite the suppressed rate of carbon fixation, *Prochlorococcus* limited for nitrogen stored more carbon and energy in the form of polysaccharides than cells grown under nutrient replete conditions (Fig. 1C), a pattern shared in N-starved freshwater cyanobacteria (28–30). Additionally, N-limited cells retained a smaller fraction of fixed carbon within the particulate (cellular) fraction (*P* = 0.15 and 0.08 for 12:00 and 16:00, respectively) (Fig. 1B), indicating pronounced losses of fixed carbon into the medium as exudate. That N-limited cells have greater release of dissolved organic carbon (DOC) and greater abundance of polysaccharides is consistent with the overflow hypothesis for carbon metabolism: carbon fixation exceeds anabolic demand due to limited N availability and thus carbon is first stored as glycogen. Once glycogen production achieves its daily quota, excess carbon can be released to the environment. Together, these results suggest nitrogen limitation places considerable constraints on carbon acquisition, and the carbon that does get fixed by the cell is redirected in major ways. The mechanisms and implications of this carbon flux rewiring are discussed later in the context of intracellular metabolite pools.

As expected, the glycogen pools in N-limited cells increased in the afternoon (*P* = 0.10) (Fig. 1C). The amount of fixed carbon retained by the cells was lower in the afternoon than at noon, indicating that even more of the carbon fixed at this time was released as exudate. Interestingly, the afternoon decline in photosynthetic rate was far less pronounced in N-limited cultures compared to the significant decline observed in N-replete cultures (Fig. 1A). The reason for this dampened response is not known but suggests that light energy is not redirected to the polymerization of macromolecules to the extent it is in N-replete cells. With the exception of glycogen, the synthesis of polymers such as DNA, RNA, and protein could be affected, which is consistent with the overall slowing of growth in N-limited cells. Effectively, this would then serve to maintain large carbon pools in the afternoon that promote elevated glycogen accumulation and exudate release. Regardless of the mechanism, these results indicate the impact of N limitation on *Prochlorococcus* carbon fixation has an important temporal context which may have important consequences for the contribution of *Prochlorococcus* to bulk CO_2_ fixation to the phytoplankton community.

### Metabolite pools and labeling rates in nitrogen-limited and -replete cultures.

The impact of nitrogen limitation on the flow of carbon though the intermediary pathways in VOL29 was addressed through measurements of metabolite pool sizes and the flux of heavy isotope carbon, originating from bicarbonate (H^13^CO_3_^−^), through the metabolite pools. In all, 52 identified metabolites were detected across treatment groups (see Table S1 in the supplemental material), of which 7 provided measurements of ^13^C flux. In most cases, pool sizes were similar for the 12:00 and 16:00 sample times (Table S1). For simplicity, metabolite data from the 12:00 sample time are presented, and instances where the time points clearly differed will be identified.

10.1128/mSystems.00254-18.4 TABLE S1 Nitrogen limited and nitrogen replete metabolite pool data. Metabolite abundance measured as the total ion counts normalized to cells per milliliter for both nitrogen (N)-limited and -replete cultures. Metabolites with observed isotopic labeling denoted with asterisks. Download Table S1, DOCX file, 0.02 MB. Copyright © 2019 Szul et al. 2019 Szul et al. This content is distributed under the terms of the Creative Commons Attribution 4.0 International license.

### Central carbon metabolites.

**FIG 2:**
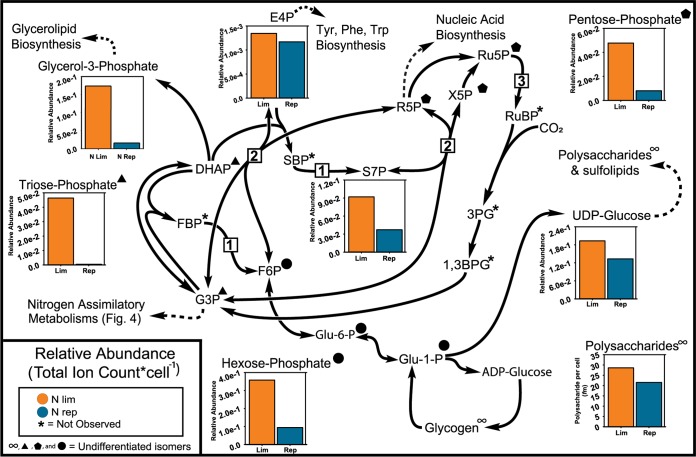
Central carbon metabolism in N-limited and -replete VOL29 cultures at 12:00. Comparisons of per cell normalized metabolite pools between nitrogen-limited and -replete cultures of VOL29 (see Table S1 in the supplemental material). Bar graphs reflect means from biological replicates. Specific enzymes addressed in discussion are fructose-1,6-bisphosphatase II/sedoheptulose-1,7-bisphosphatase (GlpX; 1), transketolase (TktA; 2), and phosphoribokinase (Prk; 3). 1,3BPG, 1,3-bisphosphoglycerate; 3PG, 3-phosphoglycerate; E4P, erythrose 4-phosphate; F6P, fructose 6-phosphate; FBP, fructose 1,6-bisphosphate; G3P, phosphoglyceraldehyde; Glu-1-P, glucose 1-phosphate; Glu-6-P, glucose 6-phosphate; R5P, ribose 5-phosphate; Ru5P, ribulose 5-phosphate; S7P, sedoheptulose 7-phosphate; SBP, sedoheptulose 1,7-bisphosphate; X5P, xylulose 5-phosphate.

*Prochlorococcus* fixes carbon dioxide with RubisCO and regenerates the RubisCO substrate RuBP via the Calvin cycle. Most Calvin cycle intermediates were detected in at least one treatment group of VOL29 (Fig. 2). Fructose bisphosphate (FBP) and sedoheptulose-P_2_ (SBP) were not detected in either nutrient treatment. Both metabolites are substrates for the unidirectional enzyme fructose-1,6-bisphosphatase II/sedoheptulose-1,7-bisphosphatase (GlpX) (Fig. 2, reaction 1). The unidirectional nature of this enzyme serves to prevent backflow in the Calvin cycle (31) and can explain the lack of detectable pools of the enzyme’s substrates. Complicating the analysis of the Calvin cycle are three sets of isomers that cannot be resolved by mass spectrometry: triose phosphate (phosphoglyceraldehyde [G3P] and dihydroxyacetone phosphate [DHAP]), hexose phosphate (fructose 6-phosphate [F6P], glucose 1-phosphate [G1P], and glucose 6-phosphate [G6P]), and pentose phosphate (xylulose 5-phosphate [X5P], ribose 5-phosphate [R5P], and ribulose 5-phosphate [Ru5P]).

Despite the ambiguity, the total pools for each of these sets of isomers, as well as the other central carbon metabolite detected, sedoheptulose-7P (S7P), were all more abundant in N-limited cells than in N-replete cells (Fig. 2). These data suggest an overall pooling of Calvin cycle intermediates when nitrogen limits the growth of *Prochlorococcus*, a trend also observed in starved cultures of green algae and freshwater cyanobacteria (28–30, 32, 33).

**FIG 3:**
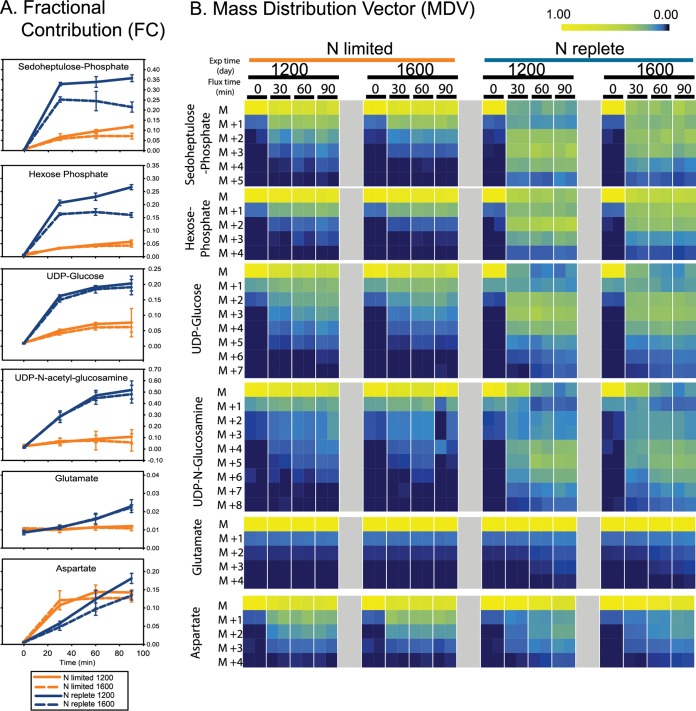
Carbon flux analysis of VOL29 N-limited and -replete cultures. Carbon flux through metabolite pools displayed through two isotopic enrichment analysis methods: fractional contribution (FC) (A) and mass distribution vector (MDV) (B). Isotopic carbon labeling of metabolite isotopologues (*M *+ *i*) is displayed as MDV, where *M* represents unlabeled isotopologues and *i* is the number of isotopically labeled atoms in the carbon backbone of the metabolite. Error bars for FC represent standard deviations. MDV data represent averages from technical replicates (*n *=* *3) for each biological replicate over 90 min. See Table S2 for IC values.

The decrease in photosynthetic carbon fixation rate (Fig. 1) and increase in Calvin cycle pool sizes (Fig. 2) during nitrogen-limited growth were concomitant with a slower flux through several Calvin cycle intermediates: S7P and the hexose phosphate isomers (Fig. 3). Fractional composition (FC) analysis (Fig. 3A), which summarizes the fraction of labeled carbon atoms in each metabolite (34), showed that relative to N-replete cells, N-limited cells had a lower initial rate of labeling and attained a lower FC value for the hexose phosphate isomers and S7P in N-replete versus N-limited cells. Metabolic labeling of N-starved cyanobacterium Arthrospira platensis showed similar results (28).

10.1128/mSystems.00254-18.5 TABLE S2 Nitrogen limited and nitrogen replete metabolite ^13^C labeling. Raw isotopologue abundance (integrated ion counts) for ^13^C-labeled metabolites in nitrogen-limited and -replete cultures at both noon (12:00) and afternoon (16:00) time points. Isotopologues are described in their unlabeled and labeled forms, where “metabolite + *i*” is the number of isotopic carbons in that isotopologue. Abbreviations: Glu, glutamate; HexP, hexose phosphate; Asp, aspartate; S7P, sedoheptulose 1/7-phosphate; UDP-Glc, UDP-glucose; UDP-GlcN, UDP-*N*-acetyl-glucosamine. Download Table S2, DOCX file, 0.03 MB. Copyright © 2019 Szul et al. 2019 Szul et al. This content is distributed under the terms of the Creative Commons Attribution 4.0 International license.

Mass distribution vector (MDV) (Fig. 3B), which describes the patterns of partial isotopic labeling for metabolites with multiple carbon atoms, shows the expected observation that metabolites accumulate isotopically labeled carbons as the Calvin cycle products (G3P) are recycled to acquire an additional heavy isotope during each passage through the RubisCO reaction. Consistent with these observations, the progression toward more heavily labeled metabolites was faster in the N-replete cultures.

Notably, while the pool sizes of S7P and hexose phosphate were not affected by the time of day, the labeling rates for these intermediates were observed to have temporal differences. In the afternoon, inorganic carbon enrichment of these metabolites in N-replete cultures reached lower levels of saturation than those observed at noon; this general trend was also observed in N-limited cells, but the effect was less pronounced (Fig. 3). This decline in afternoon flux is consistent with the decline in bulk photosynthetic carbon fixation at this later stage in the day (Fig. 1); how pool sizes remain equivalent during the change in flux is not known.

S7P and F6P serve as the substrates for transketolase (TktA) (Fig. 2, reaction 2) at two different stages of the Calvin cycle. The large pool sizes of these metabolites at noon and afternoon may serve to prevent backflow for this bidirectional enzyme during photosynthesis. As part of the pentose phosphate pathway that shares most enzymes of Calvin cycle, the reverse direction of transketolase serves a catabolic role and may be used by *Prochlorococcus* to utilize glycogen stores at night. We therefore hypothesized that as products of transketolase in the reverse direction, S7P and F6P would be depleted during the night as the cell directs carbon flow into energy-generating catabolic pathways.

Overall, it can be seen that the decrease in photosynthetic carbon fixation in N-limited cells coincides with large slow-moving pools of Calvin cycle intermediates. Although it may seem reasonable to apply rates of metabolite labeling as a measure of enzyme activity, it is important to recognize that observed labeling rates are calculated as a function of the fraction of total labeled versus unlabeled metabolite pools, and as such, the size of metabolite pools impacts the ^13^C labeling rate significantly. The mechanism by which the flux through the Calvin cycle is reduced under N limitation is not known; however, given the large pools of pentose phosphates and the absence of detectable RuBP (Fig. 2), one possible bottleneck might be the ATP-dependent phosphoribulokinase enzyme (Prk) (Fig. 2, reaction 3) that phosphorylates Ru5P to make the RuBP substrate for the RubisCO reaction.

### Lipid and cell envelope synthesis.

Pools of UDP-glucose were larger (Fig. 2) and flux through this metabolite was slower (Fig. 3) in N-limited cells than in N-replete cells. UDP-glucose is a precursor for some polysaccharides, including sucrose, a disaccharide which has been detected in several strains of *Prochlorococcus* (35). In *Prochlorococcus*, UDP-glucose may also be used for production of sulfolipids (36, 37). Sulfolipids are major components of *Prochlorococcus* cell membranes, largely replacing phospholipids as an adaptation to phosphorus limitation in the ocean. Slower flux and increased pooling of UDP-glucose may reflect the lower demand for lipids under nutrient limitation. Consistently, another prospective lipid precursor, glycerol-3-phosphate, also showed greater pooling in N-limited cultures (Fig. 2). Future studies will be needed to verify the role of UDP-glucose in polysaccharide and (sulfo)lipid production in *Prochlorococcus* and to determine how nitrogen limitation impacts each of the production pathways that stem from UDP-glucose.

UDP-*N*-acetylglucosamine (UDP-GlcNAc) serves as a precursor for peptidoglycan and lipid A biosynthesis. In replete cultures, pools of UDP-GlcNAc observed at noon diminished by almost 40% in the afternoon (Table S1). Under N-limiting conditions, metabolite pools at noon were observed at concentrations similar to those of the N-replete condition but, in contrast, no decrease in pool size was measured in the afternoon. These data suggest that under replete conditions, pools of UDP-GlcNAc form early prior to being utilized in the afternoon for *de novo* synthesis of peptidoglycan and lipopolysaccharide (LPS). These metabolites support cellular growth in the afternoon while cells prepare for cell division, which occurs in late evening or early night (38–40). In contrast, N-limited cultures have reduced demand for metabolites associated with cell growth, resulting in stagnant pools of UDP-GlcNAc.

### Nitrogen assimilation.

**FIG 4:**
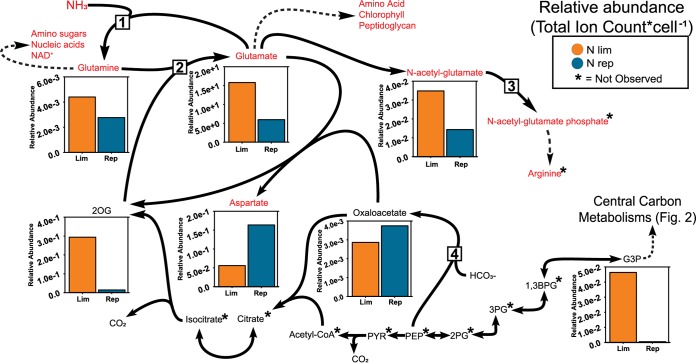
Nitrogen metabolism in N-limited and -replete VOL29 cultures at 12:00. Comparisons of per cell normalized metabolite pools between nitrogen-limited and -replete cultures of VOL29 (Table S1). Metabolites in red font contain nitrogen. Specific enzymes addressed in Discussion are glutamine synthetase (GS; 1), glutamine-oxoglutarate amido transferase (GOGAT; 2), *N*-acetyl-glutamate kinase (NAGK; 3), and phosphoenolpyruvate carboxylase (PEPc; 4). 1,3BPG, 1,3-bisphosphoglycerate; 2PG, 2-phosphoglycerate; 2OG, 2-oxoglutarate; 3PG, 3-phosphoglycerate; G3P, phosphoglyceraldehyde; OAA, oxaloacetate; PEP, phosphoenolpyruvate; PYR, pyruvate.

The tricarboxylic acid (TCA) intermediate 2OG serves as the carbon backbone for ammonia assimilation via the GS-GOGAT pathway. In N-limited VOL29, the 2OG pool was an order of magnitude larger than in N-replete cells (Fig. 4). Similar pooling of 2OG was observed in N-starved cultures of freshwater cyanobacteria and green algae (9, 14, 28, 41). This result suggests that, as for other phytoplankton, limiting the supply of the proximal N substrate for the GS-GOGAT pathway leads to a buildup of the proximal C substrate in *Prochlorococcus*. In the canonical nitrogen assimilation system, 2OG pools serve as the intracellular signal for nitrogen-limiting conditions, interacting with three cyanobacterial nitrogen regulators, namely, NtcA, PipX, and P_II_, and modulating their activity (7–10). While the responses of PipX and P_II_ to 2OG are not yet known, recent *in vitro* studies show activity of NtcA from *Prochlorococcus* is modulated in response to the 2OG concentration (12).

The aminated product of 2OG is glutamate, and this serves as a key source of organic N for multiple biosynthetic pathways. Integrated ion counts (IC) for glutamate dwarfed most other features observed, indicating that as observed in other microbial systems (42–44), *Prochlorococcus* maintains very large glutamate pools. The benefit of maintaining such large pools is poorly understood. Glutamate can serve as a compatible solute for osmoregulation, but *Prochlorococcus* has additional compatible solutes (35) and an increased need for these under nitrogen limitation seems unlikely. A mutually nonexclusive hypothesis concerns nitrogen economy and reasons that large glutamate pools potentiate the GS reaction without having to synthesize large amounts of GS enzyme (GlnA). Should ammonia become available, the high concentration of the cosubstrate of GS, glutamate, means that the amount of (nitrogen-containing) enzyme needed to effectively drive the reaction forward is reduced. This might help explain why the abundance of this enzyme in the closely related MED4 strain was not observed to increase following nitrogen starvation (17). While the product of GS, glutamine, is also present (Fig. 4), it is much lower than the substrate of GS, glutamate, and so the forward reaction of GS should still be highly favorable.

Surprisingly, both glutamate and the other N-containing intermediate of the GS-GOGAT pathway, glutamine, were found at higher concentrations in N-limited cells than in N-replete cells (Fig. 4). This contrasts with the depletion of these metabolites in N-starved cultures of green algae and freshwater cyanobacteria, as well as N-starved *Prochlorococcus* strain SS120 (28, 32, 33, 35, 41, 45). Rather than draining these pools of organic nitrogen for biosynthesis, N-limited VOL29 appears to be increasing them.

Over the 90-min flux experiment, the incorporation of the ^13^C isotope into the glutamate pool was greater in the N-replete cells than in the N-limited cells (Fig. 3A). While this rate difference may have less to do with differences in GOGAT enzyme activity and more with the much larger pool size of the N-limited cells (Fig. 4), it nonetheless indicates that relative to N-replete cells, the N-limited cells have large slow-fluxing pools of glutamate.

The increased pool sizes and decreased pool turnover of glutamate in N-limited cells could be attributed to feedback regulation of the GS-GOGAT pathway, acting at the level of enzyme synthesis and/or activity, but could also be attributed to a decreased rate of glutamate depletion by the downstream anabolic reactions. Whether or not *Prochlorococcus* tempers anabolic demand for organic nitrogen under N-limited growth conditions is a subject of future study, as is the possible regulatory mechanism(s) involved. The *Prochlorococcus* homologs of NtcA, PipX, and PII, which in other cyanobacteria play a central role in regulating intracellular C/N, are potential candidates for this metabolic regulation.

The time of day had various degrees of impact on N assimilation metabolite pools and labeling that varied as a function of metabolite and/or N status. Whereas bulk ^14^C photosynthetic rates declined in the afternoon (Fig. 1), isotopic enrichment of glutamate did not decline for N-replete or N-limited cultures (Fig. 3), suggesting that pathways involved in glutamate production are not regulated as a function of diel periodicity but by availability of nitrogen. In our N-limited cultures, we also observed pools of glutamine to grow by more than 50% during the afternoon (Table S1). We hypothesize that glutamine, an important metabolite in *de novo* nucleic acid synthesis, may pool in response to the lack of demand for new nucleotides in cells that do not replicate their chromosome in anticipation of cell division. The majority of cells in the N-limited cultures would fit this scenario, as the dilution rate of the chemostat (which in steady state is the growth rate of the population) was well below the rate for one cell doubling per day (0.09 versus 0.69 day^−1^).

### Nitrogen distribution pathways.

Consistent with the large slow-moving pools of glutamate, the route of glutamate conversion to arginine was dampened during N limitation. In addition to protein biosynthesis, arginine is a substrate for N storage metabolites such as cyanophycin or spermidine. Although neither of these storage metabolites was identified in our study, *Prochlorococcus* appears to produce spermidine (46), and a putative spermidine synthase gene (*speE*) was previously observed to be expressed with diel periodicity peaking during the late evening (25). While arginine was not detected; the precursor metabolite, *N*-acetyl-glutamate (NAG) was found in both N-replete and N-limited cells, with a 2- to 3-fold higher concentration in the latter. Conversion of NAG to arginine involves a multistep pathway that is initiated by *N*-acetyl-glutamate kinase (NAGK) (8, 11, 47). In freshwater cyanobacteria, NAGK activity is indirectly regulated by nitrogen availability and 2OG concentration: the high concentration of 2OG present in N-starved cells sequesters P_II_, while under N-replete conditions, free P_II_ protein enhances NAGK activity. The elevated pools of 2OG and NAG in our N-limited *Prochlorococcus* cells are consistent with this mechanism of regulation and suggest that for this pathway at least, 2OG and P_II_ may play equivalent roles in *Prochlorococcus* as they do in freshwater cyanobacteria.

As notable exceptions to the general trend of metabolite concentrations in VOL29, aspartate and its precursor oxaloacetate had larger pools in replete than in N-limited treatments (Fig. 4). This trend is consistent with studies of starved freshwater cyanobacteria and green algae (28, 32, 33, 45), but the mechanism may be different: these other microbes have lower glutamate pools during starvation, whereas in VOL29, the pools are larger under N limitation. Thus, while limited supplies of glutamate precursor may decrease aspartate biosynthesis in freshwater cyanobacteria and algae, this does not appear to account for the drop in aspartate pools in VOL29. N-limited cultures also had higher concentrations of G3P, which supplies the carbon backbone for oxaloacetate and aspartate (Fig. 4). Intermediates between G3P and oxaloacetate were not detected and likely consist of small pools. Thus, the limiting factor for aspartate synthesis under N limitation is not apparent from measures of detectable precursor pools.

FC analyses of aspartate in N-limited cultures showed a rapid initial uptake of isotopic label that saturated by the 30-min time point. In contrast, the large pools of aspartate in N-replete cultures incorporated label at a reduced rate initially but enriched throughout the 90-min time series (Fig. 3A), again pointing to differences in regulation of this pathway under these different physiological conditions.

In N-replete cultures, the rate of ^13^C labeling of aspartate was greater at noon than in the afternoon (Fig. 3A). Decreased flux through aspartate in the afternoon could reflect the role of aspartate as a precursor in the synthesis of nucleic acid components: pyrimidines. Pyrimidine production and incorporation into nucleotide monomers may be maximal at noon, in anticipation of chromosomal replication in the late afternoon (25). Hence, a fast flux through aspartate at noon would reflect a metabolic emphasis on building the monomers, and a slower flux in the afternoon would reflect a decreased demand for monomers and a shift toward polymerization of the pooled monomers. Notably, in N-limited cultures, the ^13^C enrichment was the same at noon and in the afternoon (Fig. 3A), and this may reflect an overall lower demand for nucleotides under conditions where cells are not rapidly dividing.

Without an intact TCA cycle (48), the pathway for *de novo* synthesis of aspartate in *Prochlorococcus* spp. occurs through the condensation of bicarbonate, HCO_3_^−^, and the three-carbon metabolite phosphoenolpyruvate (PEP) by phosphoenolpyruvate carboxylase (PEPc). Aspartate is generated when the amino group of glutamate is transferred to the product of the PEPc reaction, oxaloacetate (OAA). This process generates the four-carbon metabolite, aspartate, where three of the four carbon atoms are fixed via the Calvin cycle and the fourth is fixed by PEPc (Fig. 4). The observation of increasingly heavy, i.e., ^13^C-labeled, isotopologues (MDV) of aspartate under N-replete conditions provides evidence for the ongoing labeling of precursor metabolites (i.e., PEP) via the Calvin cycle (Fig. 3B). In our N-limited cultures, we observed heavy aspartate isotopologues after the first time point, but FC reached steady state and the MDV analyses showed isotopic labeling in the isotopologue did not become increasingly enriched. This observation may suggest that by doubling the concentration of bicarbonate in the medium through the addition of our radiolabeled tracer, Na^13^CO_3_^−^, the activity of PEPc was stimulated. This increased activity could be the mechanism for our observation of immediate but unsustained production of aspartate. This observation, along with the experimental metabolic restrictions, raises the hypothesis that PEPc and the synthesis of aspartate may be under indirect control of the P_II_ regulator. Our hypothesis is supported by data which suggest PEPc is sensitive to the concentration of intracellular bicarbonate and a previous report that showed evidence that the bicarbonate transporter is under the control of P_II_ in *Prochlorococcus* PCC 9511 (15).

### Field metabolite analysis.

**FIG 5:**
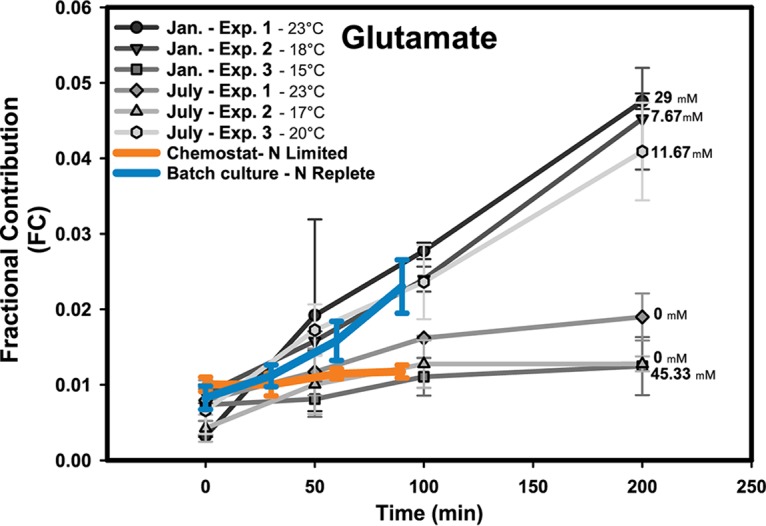
Glutamate flux in natural populations of *Prochlorococcus* in the North Pacific Ocean. Over a series of two research cruises, six experiments measuring carbon flux through metabolites in *Prochlorococcus*-enriched microcosms were measured. Comparisons of FC analysis of cruise data (grayscale lines) and the laboratory nitrogen limited and nutrient replete (orange and blue lines, respectively). Sea surface temperatures for each station are noted in the legend, and measurements of ammonium concentrations are noted at the end of the line that they describe (Table S3).

Several lines of evidence suggest that natural *Prochlorococcus* populations are limited by nitrogen in the surface mixed layer of the North Pacific Ocean (3–5). We performed an initial metabolomics analysis of *Prochlorococcus* in the North Pacific in January and July 2013 (see Table S3) to determine if patterns of carbon flux were more consistent with N-limited or N-replete VOL29 cultures. Metabolism of *in vivo Prochlorococcus* was assessed by taking advantage of its unique status as the smallest known free-living photosynthetic organism. In microcosms containing the size-fractionated <0.8-μm microbial community, *Prochlorococcus* spp. are the only populations capable of photosynthetic carbon fixation, and as such, they are the only organisms capable of fixing the significant amounts of ^13^C-labeled inorganic carbon observed in our measurements. An important caveat to the FC measurement is that the total metabolite pool (^12^C plus ^13^C) reflects the entire community that passed through the 0.8-μm filter, including heterotrophs. Fewer metabolites were detected in this initial labeling experiment than in the laboratory culture experiment, likely due to the low *Prochlorococcus* biomass at these sites. However, the labeling profiles of glutamate reflect the labeling trend reported in the laboratory when environmental conditions were considered (Fig. 5). At stations where the NH_4_^+^ concentration was above the limit of detection, a high rate of carbon flux into glutamate was found for *Prochlorococcus* cells, analogous to the high rates observed for N-replete cultures. In contrast, when *in situ* NH_4_^+^ concentrations were below the limit of detection, flux was much slower, analogous to the flux of the laboratory N-limited chemostat cultures. The lone exception to these observations was recorded during the January experiment 3 (Exp3), where NH_4_^+^ was observed at high concentrations (∼45 nM) but carbon flux was not seen in *Prochlorococcus*-enriched microcosms. However, the surface seawater temperature was 15°C, lower than the other stations by at least 2°C, and critically, the concentration of *Prochlorococcus* was only approximately 2-fold lower at this station (Table S3). Thus, the flux of carbon through glutamate could have been masked by low cell density and perhaps lower enzymatic rates that likely occur at the lower temperature.

10.1128/mSystems.00254-18.6 TABLE S3 Experimental station details. Experiments and sampling occurred over two similar cruise tracks separated by approximately 6 months in the North Pacific Ocean. Cell counts performed on whole water samples prior to size fractionation. Download Table S3, DOCX file, 0.01 MB. Copyright © 2019 Szul et al. 2019 Szul et al. This content is distributed under the terms of the Creative Commons Attribution 4.0 International license.

### Conclusions.

In this study, we report comparisons of physiological and metabolic analyses for *Prochlorococcus* and provide for new insights into the underlying mechanisms of photosynthesis under nitrogen limitation, the predominant state of this genus in a large fraction of the ocean (3–5). As observed in other phytoplanktonic systems (28, 29, 49), nitrogen limitation during the growth of *Prochlorococcus* VOL29 reduces photosynthetic carbon fixation rates while increasing polysaccharide stores. This apparent conserved physiological response to nitrogen limitation suggests shared underlying dynamics of nitrogen deficiency on photosynthetic organisms, but the mechanisms remain poorly understood.

Our data show nitrogen limitation in *Prochlorococcus* generates large stagnant metabolite pools compared to the small pools with high turnover observed in our nitrogen replete experiments. In addition to the large pools of glutamate, nitrogenous metabolites, including inosine and UDP*-N*-acetylglucosamine, were observed to pool under N-limiting conditions. Whereas other N-limited phytoplankton drain their pools of nitrogen-containing metabolites (28, 32, 33, 35, 41, 45), *Prochlorococcus* strain VOL29 appears to largely do the opposite and expand these pools. While only speculation at this point, we hypothesize that the N limitation bottleneck in the biosynthesis of *Prochlorococcus* cells is found downstream of glutamate and glutamine, perhaps at the step of transamination (for biosynthesis of other amino acids), the later stages of nucleic acid biosynthesis, chromosome replication, transcription, and/or translation. How these processes are impacted by N limitation, either passively or via regulatory control, is unknown and awaits future study. One possible mechanism contributing to pool expansion was recently reported in the related *Prochlorococcus* strain (MED4). In that study, the authors demonstrated that N-deprived MED4 generated shortened transcripts that resulted in truncated proteins that are thought to retain functionality (50). Reducing the demand for amino acids and their precursors may serve as one mechanism that generates large slow-moving pools under nitrogen limitation.

While both metabolite pools and the flow of labeled inorganic carbon into these metabolites were quantified, it has become clear that future investigations will be required to ascertain if such differences were due to passive effects or if there is regulatory control (e.g., allosteric feedback inhibition, expression regulation, or posttranslational modification) at play for the anabolic pathways analyzed. Further investigations of the effect of nutrient limitation throughout the diel are warranted, given the observation that N limitation dampens the difference in flux and pool sizes at noon versus afternoon for some (bulk photosynthesis, glutamate, and aspartate) but not all (glycogen and exudate release) metabolic features of *Prochlorococcus*. This finding also highlights the need for considering local time of day when performing physiological measurements of phytoplankton in natural, often nutrient limiting, conditions.

## MATERIALS AND METHODS

### Culture and incubator conditions.

Axenic *Prochlorococcus* sp. strain VOL29, a member of the eMED4 ecotype isolated in the North Pacific (9 March 2012, 29°6′N and 125°07′W, collected at a 3-m depth, 15.7°C) during POWOW 1 (24), was grown from a master culture maintained in serially passaged batch cultures for greater than 6 months under experimental conditions: AMP-A medium, 22°C, 14 h of gradually increasing and decreasing light levels designed to mimic natural levels of light over the course of the day (see Fig. S2 in the supplemental material). AMP-A is an autoclaved artificial seawater medium derived from AMP1 (51) where the only difference is that (NH_4_)_2_SO_4_ (400 μM) has been replaced with NH_4_Cl (800 μM). Sunrise in the incubator occurred at 04:00, with peak light levels at 11:00 and sunset at 18:00. Cultures were sampled for metabolomics and other analyses at 12:00 and 16:00. Prior to beginning and following our experiments, all cultures were tested for absence of heterotrophic contamination by lack of turbid growth for more than 1 month in 3 media of various nutrient richness (PLAG, 1/10 ProAC, and yeast-tryptone-sea salts [YTSS] [23]). Culture cell concentration was measured using forward scatter and red autofluorescence on a Guava easyCyte 5HT (EMD Millipore, Burlington, MA).

Prior to the experiment, N-replete batch cultures were transferred from 20-ml test tubes in AMP-A to successively larger bulk culture vessels containing modified AMP-A medium (5 mM Tris-acetate-phosphates [TAPs]) and slowly stirred with a Teflon-coated magnetic stir bar. Four days prior to the experiment (day −4) (Fig. S1), 0.2 liters culture was transferred from a single 2-liter Pyrex bottle (06-414-1E; Corning, Corning, NY) to four identical bottles containing 2.0 liters medium (2.2 liters final volume) and incubated for 4 days to reach log phase growth (μ ≈ 0.44); the experiment then began on day 0 (Fig. S1) when culture concentrations were near those of known steady-state concentration of chemostat (Fig. S3).

10.1128/mSystems.00254-18.1 FIG S1 N-limited and N-replete cultures prior to and following experiments. Comparisons of VOL29 growth under nitrogen-limited (Nlim; orange) and -replete (Nrep; blue) growth conditions leading up to and following destructive sampling of chemostats on the day of the experiment (day 0; dotted vertical line). After day 0, nitrogen amendment experiments (day 1 to 10) were performed in batch culture (+N) and compared to unamended controls (Cntrl). Mean per cell fluorescence, as determined by the average fluorescence of events counted by flow cytometry, allows for the comparison of the average chlorophyll content per cell. Error bars represent standard deviations. Download FIG S1, JPG file, 0.5 MB. Copyright © 2019 Szul et al. 2019 Szul et al. This content is distributed under the terms of the Creative Commons Attribution 4.0 International license.

10.1128/mSystems.00254-18.2 FIG S2 Light intensity of sunbox incubator. Light levels designed to mimic pattern of natural sunlight shown as white on black background. Artificial sunrise occurred at 04:00 and sunset at 18:00. Experimental time points began at 12:00 and 16:00. Download FIG S2, JPG file, 0.3 MB. Copyright © 2019 Szul et al. 2019 Szul et al. This content is distributed under the terms of the Creative Commons Attribution 4.0 International license.

10.1128/mSystems.00254-18.3 FIG S3 Comparison of VOL29 growth in current and trial grow-out experiments. Under identical growth conditions to our current study (red lines), VOL29 cultures grown in N-limited chemostats were observed to stabilize around 4.5 × 10^7^ cells · ml^−1^ over a 15-day grow-out experiment (black lines), suggesting that growth rates for the conditions tested are approximately balanced to the dilution rate of the chemostat (∼0.09 day^−1^). The 7-day period shown for the current study correspond to days −6 through 0 for Fig. 1. Download FIG S3, JPG file, 0.6 MB. Copyright © 2019 Szul et al. 2019 Szul et al. This content is distributed under the terms of the Creative Commons Attribution 4.0 International license.

N-limited cultures were generated through the use of a chemostat. Briefly, 400 ml of late-log-phase N-replete batch culture was pelleted, resuspended in 100 ml N-free basal medium (Turks Island Salt Mix [51]), and divided evenly to four 2-liter Pyrex bottles containing approximately 2-liters modified N-limited AMP-A (5 mM TAPs, 16 μM NH_4_Cl). To achieve N-limited growth, cultures were grown for 4 days prior to initiating the pump (Sci-Q 323S; Watson Marlow, Wilmington, MA). At chemostat start (day −6) (Fig. S1), the system was initially primed at a high flow rate prior to dropping the flow rate to a constant rate, replacing ∼9% of culture volume daily. Platinum-cured silicone tubing (EW-96410-14; Cole-Palmer, Vernon Hills, IL), Omnifit “T” series 3-port bottle caps (00945T3; Watson-Marlow, Wilmington, MA), manifold tubing (981.0076.000; Watson-Marlow, Wilmington, MA), and containers contacting culture media were acid washed. Cultures of N-limited *Prochlorococcus* VOL29 cells were destructively sampled 6 days after chemostat dilution rates (∼0.09 day^-1^) were set. At this time, chemostat cultures stabilized near concentrations observed in pilot continuous cultures (Fig. S3).

To minimize environmental differences between batch and continuous cultures, all possible variables, including culture vessels, stir speed, and light conditions, were maintained between experiments. Excluding those of the experimental design, the most significant difference between culture conditions was use of 3-port bottle caps on chemostat cultures while batch cultures utilized standard bottle caps (GL 45; Fisher Scientific, Hampton, NH). The 3-port cap allows for sterile passage of plastic tubing through the cap for fresh medium input, culture outflow, and culture sample collection. Chemostat culture daily cell concentration measurements were made via syringe aspiration of a small sample (<0.5 ml) through the above-noted culture sample collection port, while batch cultures were briefly removed from the incubator and taken to a sterile hood where equivalent-sized samples were aspirated for measurements. In addition to these differences, the rigid tubing for both fresh medium input and culture sample collection were below the surface of the culture broth, causing a slight perturbation in stirring and increase of cell contact with plastic surfaces.

The state of N limitation for both treatments was tested following the destructive sampling of cultures through additions of 800 μM NH_4_Cl to 5 ml batch subcultures (days 1 to 10) (Fig. S1). When N-limited batch subcultures were provided NH_4_^+^, they were observed to enter log-phase growth and the per cell fluorescence was observed to increase to levels seen in replete cultures, while cultures that did not receive NH_4_^+^ additions were not rescued. Additions of NH_4_^+^ to batch subcultures from replete master cultures were not observed to affect either growth rate or per cell fluorescence.

For both treatments, growth cultures were sampled at two time points, 12:00 and 16:00, in order to observe the role of cell cycle on metabolism. At each time point, replicate 2-liter cultures were removed from the incubator and destructively sampled.

### Photosynthetic rates.

Photosynthetic inorganic carbon fixation was measured for total organic carbon fixation rates (TOC) and the particulate organic carbon fixation rates (≥0.2 μm; POC) as previously described (25). Briefly, for each culture, two sets of triplicate 2-ml cultures were spiked with 10 μl (10 μCi) NaH^14^CO_3_ (MP Biomedicals, Santa Ana, CA) and placed in the incubator. One set of each was covered with aluminum foil and incubated under aphotic conditions. Total photosynthetic rates were calculated by subtracting rates observed in the dark (measured ^14^C due to absorption and aphotic carbon fixation) from the total photosynthetic rate observed under photic conditions.

### Lab metabolomics sample collection.

Five hundred fifty milliliters of culture from each 2-liter culture flask was transferred to 1-liter Pyrex bottles containing a Teflon-coated magnetic stir bar and enough ^13^C sodium bicarbonate to raise culture concentrations to 0.6 mM over ambient levels (standard AMP medium starts at 0.6 mM). Cultures were immediately returned to the incubator and sampled for flux measurements at 30, 60, and 90 min. While the label experiment was being moved to the incubator, time zero measurements were collected using unlabeled culture from the parent culture. For each sample, 150 ml of culture was collected using high-vacuum filtration on 0.2-μm Isopore filters (Millipore, St. Louis, MO); the filters were flash frozen in liquid nitrogen and stored at −150°C until extraction.

### Field metabolomics sample collection.

Seawater was collected from the mixed layer of the oligotrophic North Pacific Ocean in January and July of 2013 (POWOW 2 and POWOW 3, KM1301 and KM1312, respectively) predawn by CTD-Niskin rosette. Once the water was aboard the ship, it was size fractionated through gentle (gravity during POWOW 2 and low positive pressure during POWOW 3) 0.8-μm filtration, thereby enriching phytoplankton communities for *Prochlorococcus*. Acid-washed 250-ml polycarbonate bottles were filled to completion (∼300 ml) with the *Prochlorococcus*-enriched filtrate. Elimination of air bubbles by displacement with filtrate was performed to reduce the effects of turbulence and dissolved gasses on microcosms (i.e., bottle effects). Microcosms were placed in a deck-board, UV-opaque, surface-sea-temperature flowthrough incubator shaded with a single layer of 25% transmission Roscolux number 98 medium gray filter (Rosco, Stamford, CT) to acclimate for 2 to 3 h. Shortly after noon, time zero samples were collected and the remaining samples were spiked with isotopically labeled inorganic carbon (0.6 mM NaH^13^CO_3_) for flux measurements at 50, 100, and 200 min. At each time point, biological replicate samples were collected. For each sample, 0.6 liters (two full bottles) was removed from the incubator, collected via high pressure filtration on a 0.2-μm Magna nylon filter (GVS, Morecambe, United Kingdom), flash frozen, and stored in liquid nitrogen (LN_2_) at −150°C in a deep freezer prior to sample extraction in the laboratory. A FACSCalibur flow cytometer (Becton Dickinson, Franklin Lakes, NJ) was used to determine phytoplankton densities as previously described (52).

### Extraction of metabolites from filters.

Filters were defrosted, and the cells washed from the filters in 1.3 ml of extraction solvent (40:40:20 high-pressure liquid chromatography[HPLC]-grade methanol, acetonitrile, water with 0.1% formic acid) and kept at 4°C in 1.5-ml centrifuge tubes. The extraction proceeded for 20 min at −20°C, after which the samples were centrifuged for 5 min (16.1 relative centrifugal force [rcf]) at 4°C. The supernatants were then transferred to new vials and the extraction supernatants were dried under a stream of N_2_ until the extracted metabolites were a solid residue. This residue was resuspended in 300 µl of sterile water and transferred to 300-µl autosampler vials. Samples were immediately placed in autosampler trays for mass spectrometric analysis.

### Ultraperformance liquid chromatography-mass spectrometry analysis.

Samples were kept at 4°C in the autosampler until analysis. A 10-µl aliquot was injected through a Synergi 2.5-μm Hydro-RP 100, 100 mm by 2.00 mm LC column (Phenomenex, Torrance, CA) kept at 25°C. The metabolites were then eluted using a gradient consisting of two solvents with a total flow rate of 200 µl/min. Solvent A consisted of 97:3 water-methanol, 10 mM tributylamine, and 15 mM acetic acid. Solvent B was methanol. The gradient consisted of the following solvent mixtures and times: from 0 to 5 min the eluent was 100% A-0% B, from 5 to 13 min the eluent switched to 80% A-20% B, from 13 to 15.5 min the eluent was 45% A-55% B, from 15.5 to 19 min the mixture was 5% A-95% B, and from 19 to 25 min the eluent returned to 100% A-0% B. The mass spectrometer was run in full-scan mode with negative ionization using a previously described method (53). The eluent was introduced into the mass spectrometer via an electrospray ionization source coupled to a Thermo Scientific Exactive Plus Orbitrap mass spectrometer through a 0.1-mm-internal-diameter fused silica capillary tube. The samples were run with a spray voltage of 3 kV, nitrogen sheath gas flow rate of 10 (unitless), a capillary temperature of 320°C, and an automatic gain control (AGC) target set to 3e6. The samples were analyzed with a resolution of 140,000 and a scan window of 85 to 800 *m/z* from 0 to 9 min and 110 to 1,000 *m/z* from 9 to 25 min.

### Data processing.

Files generated by Xcalibur (RAW) were converted to the open-source mzML format (54) via the open-source ProteoWizard package (55). The MAVEN software platform (56, 57) was used to align and automatically correct the total ion chromatograms based on the retention times for each sample. Known metabolites were manually selected using ±5 ppm *m/z* and 30-s retention time windows for each analyte. Total ion counts were determined by manually integrating chromatogram peaks for each metabolite. To facilitate comparisons of metabolite abundances across samples with different culture concentrations, the relative abundance of each metabolite was calculated as total ion counts of that metabolite normalized to cells per sample (relative abundance = total ion count · cell^−1^) (Table S1).

The mass distributed vector (MDV) (equation 1) and fractional contribution (FC) (equation 2) analyses are useful tools to interpret labeling in metabolomics experiments and have been described previously (34). Data used to calculate both MDV and FC are provided in Table S2. The MDV is a method to measure the fractional abundance of each isotopologue to the sum of all isotopologues of that metabolite. This method describes the distribution of labeled isotopologues for each metabolite. Fractional contribution (FC) may be used to develop a better understanding the abundance of isotopic label within each metabolite over an experiment. This can be determined by calculating ratio of the relative number of labeled carbon atoms (relative labeled carbon [RLC]) to the total relative number of carbon atoms in that metabolite (relative total carbon [RTC]) [equation 2].

(1) $$\text{Mass distribution vector (MDV)}=\frac{\text{IC}}{\text{TIC}}=\frac{{\text{IC}}_{\left(M+i\right)}}{\sum_{\left(i=0\right)}^{c}\left({\text{IC}}_{\left(M+i\right)}\right)}$$

(2) $$\text{Fractional contribution (FC)}=\frac{\text{RLC}}{\text{RTC}}=\frac{\sum_{i=1}^{c}\left({\text{IC}}_{\left(M+i\right)}\times i\right)}{c\sum_{\left(i=0\right)}^{c}\left({\text{IC}}_{\left(M+i\right)}\right)}$$

The RTC per metabolite was quantified by summing integrated ion count peaks for each isotopologue (IC_(_*_M+i_*_)_) specific to that metabolite, the same as total ion count (TIC) in equation 1. The TIC is scaled to the total number of carbon atoms in the metabolite (*c*) to get the RTC. Next, the relative abundance of isotopically labeled carbon associated with that metabolite is determined (RLC). This was calculated as the sum of the product of isotopologues, IC_(_*_M+i_*_)_, and the number of labeled carbon atoms in the metabolite (*i*). Heatmaps were then generated from the data using the Java Treeview (58) software package. Note that values have not been corrected for natural abundance of ^13^C. Natural abundance can be observed as partial labeling at time zero.

### Polysaccharide pool size.

**(i) Sample collection, extraction, and precipitation of glycogen.** Biological replicates were measured in triplicates. For each of the measurements, 300 ml was collected via centrifugation (15 min at 15,000 × *g* with half-speed acceleration and deceleration) and resuspended in 1 ml fresh AMP. Glycogen extraction and measurement were performed as previously described (59). Briefly, 0.5 ml resuspended pellet was flash frozen in LN_2_ and stored at −80°C until the samples were extracted. The frozen culture was freeze-dried, resuspended in 0.5 ml 30% KOH, and vortexed vigorously for approximately 20 mins. Following resuspension, the samples were placed in a boiling water bath for 4 h with intermittent shaking and then allowed to cool to room temperature until the next step.

Glycogen, which is resistant to alkali environments of the extraction protocol, was then purified and precipitated through the stepwise additions of Milli-Q water (1 ml) and absolute ethanol (2.77 ml). Glycogen was pelleted by centrifugation at 10,000 rcf for 15 mins. The pellet was washed in 1 ml ice-cold 60% ethanol (EtOH), the supernatant was aspirated, and the pellet was vacuum desiccated for quantification. Desiccated samples were resuspended in 0.25 ml Milli-Q water and transferred to an Eppendorf tube containing 0.25 ml 5% phenol solution. After the samples were vortexed, 1 ml concentrated sulfuric acid was added, hydrolyzing the glycogen to glucose. Samples were cooled to room temperature for approximately 15 min prior to their measurement. Glycogen concentration was determined through the measurement of absorbance at 488 nm on a Beckman Coulter DU 800 spectrophotometer. The linear regression of glucose standards at 250, 100, 75, 50, 25, and 10 mg/ml in 0.15% benzoic acid provides estimates of glucose associated with the glycogen macromolecules.
