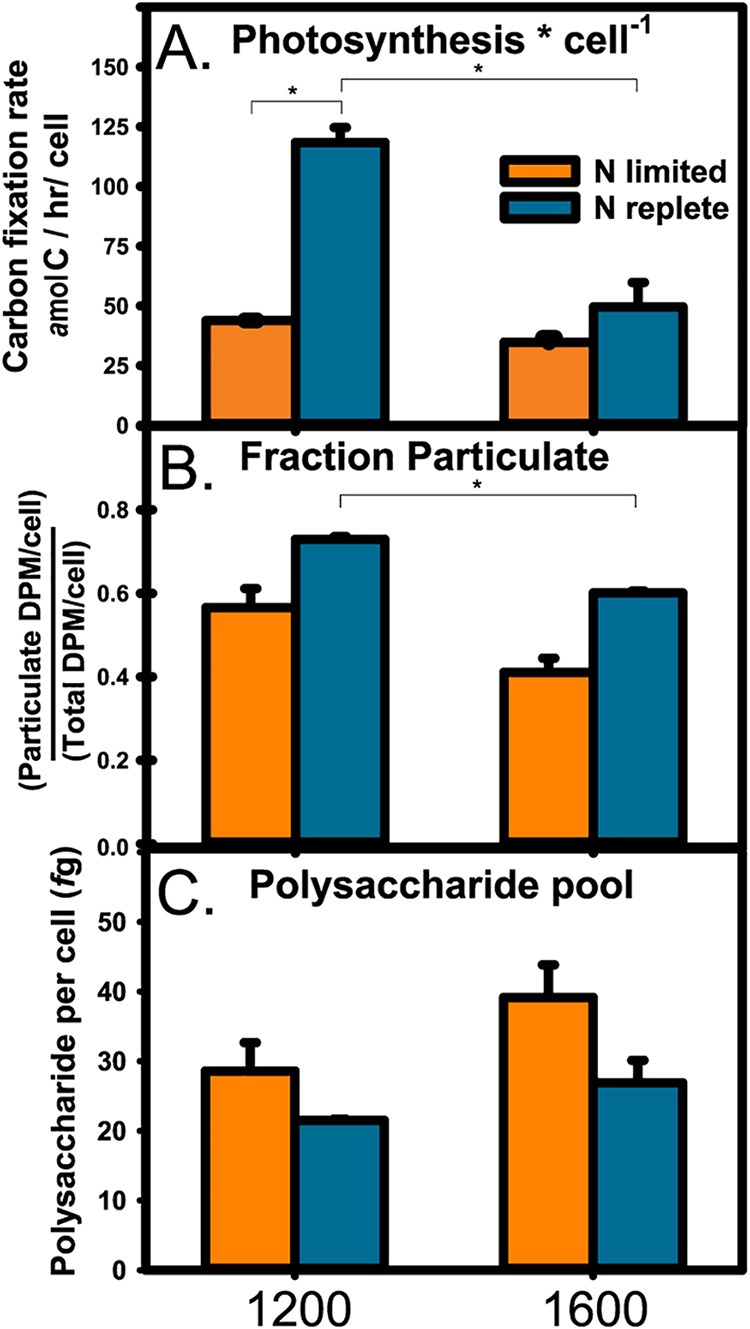# Correction for Szul et al., “Carbon Fate and Flux in *Prochlorococcus* under Nitrogen Limitation”

**DOI:** 10.1128/mSystems.00204-19

**Published:** 2019-04-02

**Authors:** Martin J. Szul, Stephen P. Dearth, Shawn R. Campagna, Erik R. Zinser

**Affiliations:** a Department of Microbiology, University of Tennessee, Knoxville, Tennessee, USA; b Department of Microbiology and Immunology, College of Graduate Studies, Midwestern University, Downers Grove, Illinois, USA; c Department of Chemistry, University of Tennessee, Knoxville, Tennessee, USA; d Biological and Small Molecule Mass Spectrometry Core, University of Tennessee, Knoxville, Tennessee, USA

## AUTHOR CORRECTION

Volume 4, no. 1, e00254-18, 2019, https://doi.org/10.1128/mSystems.00254-18. Page 4: In Fig. 1A, the unit of measurement on the *y* axis should be as shown here.

**FIG 1:**