# Supplementary material for: Carbon Fate and Flux in Prochlorococcus under Nitrogen Limitation
Source: mSystems. 2019 Feb 26;4(1):e00254-18. doi: 10.1128/mSystems.00254-18 (PMC6392094; doi:10.1128/mSystems.00254-18)
Supplement: TABLE S1 [file mSystems.00254-18-st001.docx]

|  |  | Ion Count (IC) per cell | | | |
| --- | --- | --- | --- | --- | --- |
|  |  | N-limited | | N-replete | |
| Observed Metabolite | Abbreviations  used in text | 1200 | 1600 | 1200 | 1600 |
| 2-keto-isovalerate |  | 1.163 | 0.973 | 1.668 | 1.030 |
| 4-aminobutyrate |  | 0.029 | 0.024 | 0.021 | 0.014 |
| 6-phospho-d-gluconate |  | 0.056 | 0.055 | 0.076 | 0.058 |
| 2-oxoglutarate | 2OG | 0.294 | 0.192 | 0.014 | 0.010 |
| AMP |  | 0.046 | 0.038 | 0.028 | 0.022 |
| Aspartate* |  | 0.056 | 0.056 | 0.178 | 0.160 |
| Citraconic acid |  | 0.773 | 0.611 | 0.937 | 0.736 |
| CMP |  | 0.062 | 0.061 | 0.021 | 0.014 |
| dAMP |  | 0.003 | 0.003 | 0.004 | 0.002 |
| Deoxyinosine |  | 0.000 | 0.001 | 0.005 | 0.001 |
| Deoxyribose-phosphate |  | 0.055 | 0.053 | 0.003 | 0.002 |
| Erythrose-4-phosphate | E4P | 0.001 | 0.001 | 0.001 | 0.001 |
| Fumarate |  | 0.006 | 0.005 | 0.007 | 0.006 |
| Gluconate |  | 0.013 | 0.014 | 0.009 | 0.005 |
| Hexose-phosphate* | HexP | 0.358 | 0.303 | 0.100 | 0.091 |
| Glutamate* | Glu | 15.803 | 12.912 | 6.387 | 5.117 |
| Glutamine | Gln | 0.003 | 0.006 | 0.002 | 0.002 |
| Glutathione |  | 0.168 | 0.206 | 0.096 | 0.070 |
| glutathione disulfide |  | 0.189 | 0.142 | 0.074 | 0.058 |
| glyceraldehdye-3-phosphate | G3P | 0.046 | 0.044 | 0.001 | 0.000 |
| Hydroxyisocaproic acid |  | 0.197 | 0.175 | 0.292 | 0.178 |
| Hydroxyphenylacetic acid |  | 0.015 | 0.012 | 0.025 | 0.015 |
| Hydroxyphenylpyruvate |  | 0.034 | 0.095 | 0.112 | 0.062 |
| Inosine |  | 3.048 | 2.596 | 1.818 | 1.618 |
| Leucine/isoleucine |  | 0.002 | 0.002 | 0.001 | 0.001 |
| Malate |  | 0.012 | 0.012 | 0.016 | 0.011 |
| Methylmalonic acid |  | 0.420 | 0.338 | 0.525 | 0.360 |
| *N*-acetylglutamate | NAG | 0.035 | 0.024 | 0.014 | 0.015 |
| *N*-Acetyltaurine |  | 0.000 | 0.002 | 0.001 | 0.000 |
| NAD+ |  | 0.001 | 0.001 | 0.001 | 0.000 |
| Oxaloacetate |  | 0.003 | 0.002 | 0.004 | 0.004 |
| Phenylalanine |  | 0.004 | 0.003 | 0.007 | 0.003 |
| *p*-hydroxybenzoate |  | 0.021 | 0.017 | 0.030 | 0.018 |
| Proline |  | 0.001 | 0.002 | 0.003 | 0.001 |
| Pyroglutamic acid |  | 0.031 | 0.022 | 0.077 | 0.021 |
| Pentose-phosphate |  | 0.048 | 0.044 | 0.009 | 0.005 |
| Sedoheptulose-1/7-phosphate* | S7P | 0.092 | 0.069 | 0.039 | 0.029 |
| Serine |  | 0.003 | 0.003 | 0.002 | 0.001 |
| *sn*-glycerol-3-phosphate |  | 0.174 | 0.154 | 0.016 | 0.015 |
| Sulfolactate |  | 0.026 | 0.023 | 0.031 | 0.026 |
| Threonine |  | 0.002 | 0.003 | 0.002 | 0.001 |
| Trehalose/Sucrose |  | 0.006 | 0.012 | 0.009 | 0.007 |
| Tryptophan |  | 0.001 | 0.001 | 0.001 | 0.000 |
| Tyrosine |  | 0.100 | 0.123 | 0.010 | 0.005 |
| UDP-d-glucose* | UDP-glu | 0.193 | 0.188 | 0.136 | 0.081 |
| UDP-*N*-acetylglucosamine* | UDP-GlcNAc | 0.069 | 0.069 | 0.072 | 0.044 |
| UMP |  | 0.012 | 0.019 | 0.014 | 0.004 |
| Unknown_C5H4N4O2 |  | 0.054 | 0.039 | 0.069 | 0.054 |
| Uric acid |  | 0.001 | 0.004 | 0.013 | 0.001 |
| Uridine |  | 0.001 | 0.002 | 0.003 | 0.002 |
| Valine |  | 0.004 | 0.004 | 0.001 | 0.000 |
| Xanthine |  | 0.000 | 0.001 | 0.000 | 0.000 |
