# Supplementary material for: Carbon Fate and Flux in Prochlorococcus under Nitrogen Limitation
Source: mSystems. 2019 Feb 26;4(1):e00254-18. doi: 10.1128/mSystems.00254-18 (PMC6392094; doi:10.1128/mSystems.00254-18)
Supplement: TABLE S2 [file mSystems.00254-18-st002.docx]

|  | **Nitrogen Limited** | | | | | | | | | | | | | | | |
| --- | --- | --- | --- | --- | --- | --- | --- | --- | --- | --- | --- | --- | --- | --- | --- | --- |
| metabolite isotopologue | 1200 | | | | | | | | 1600 | | | | | | | |
|  | 0 | | 30 | | 60 | | 90 | | 0 | | 30 | | 60 | | 90 | |
| Glu | 5.5E+8 | 9.1E+8 | 5.6E+8 | 6.0E+8 | 7.6E+8 | 6.8E+8 | 7.7E+8 | 9.3E+8 | 7.6E+8 | 5.0E+8 | 3.9E+8 | 4.1E+8 | 6.3E+8 | 5.4E+8 | 4.3E+8 | 4.0E+8 |
| Glu +1 | 2.3E+7 | 4.4E+7 | 2.4E+7 | 3.0E+7 | 3.8E+7 | 3.8E+7 | 4.1E+7 | 5.3E+7 | 3.8E+7 | 2.4E+7 | 1.9E+7 | 2.0E+7 | 3.3E+7 | 2.9E+7 | 1.9E+7 | 2.1E+7 |
| Glu +2 | 1.9E+6 | 3.6E+6 | 1.7E+6 | 2.0E+6 | 2.6E+6 | 2.2E+6 | 2.6E+6 | 3.2E+6 | 3.0E+6 | 1.8E+6 | 1.2E+6 | 1.4E+6 | 2.1E+6 | 1.9E+6 | 1.4E+6 | 1.3E+6 |
| Glu +3 | 1.9E+4 | 7.9E+4 | 5.3E+4 | 1.5E+5 | 2.1E+5 | 3.4E+5 | 2.9E+5 | 5.7E+5 | 9.2E+4 | 1.8E+4 | 1.1E+5 | 6.6E+4 | 3.2E+5 | 2.6E+5 | 2.5E+5 | 2.4E+5 |
| Glu +4 | 1.0E+3 | 3.1E+4 | 6.5E+3 | 2.4E+4 | 3.7E+4 | 4.6E+4 | 3.6E+4 | 8.8E+4 | 2.0E+4 | 1.9E+3 | 1.3E+4 | 5.1E+3 | 3.4E+4 | 2.2E+4 | 2.9E+4 | 4.0E+4 |
|  |  |  |  |  |  |  |  |  |  |  |  |  |  |  |  |  |
| HexP | 1.4E+7 | 1.9E+7 | 8.4E+6 | 8.2E+6 | 9.5E+6 | 9.7E+6 | 7.1E+6 | 9.3E+6 | 1.9E+7 | 1.1E+7 | 8.2E+6 | 6.2E+6 | 5.4E+6 | 6.0E+6 | 5.7E+6 | 4.1E+6 |
| HexP+1 | 5.9E+5 | 8.8E+5 | 1.7E+6 | 1.6E+6 | 2.4E+6 | 2.4E+6 | 2.1E+6 | 2.4E+6 | 8.9E+5 | 4.4E+5 | 1.8E+6 | 1.3E+6 | 1.4E+6 | 1.5E+6 | 1.6E+6 | 1.0E+6 |
| HexP+2 | 1.1E+3 | 1.1E+4 | 1.8E+5 | 1.1E+5 | 4.5E+5 | 3.3E+5 | 5.6E+5 | 4.6E+5 | 6.3E+3 | 3.0E+3 | 1.2E+5 | 7.2E+4 | 1.5E+5 | 1.8E+5 | 1.5E+5 | 1.2E+5 |
| HexP+3 | 3.8E+3 | 1.6E+3 | 2.1E+4 | 6.6E+3 | 1.0E+5 | 4.0E+4 | 1.4E+5 | 1.0E+5 | 1.7E+3 | 0.0E+0 | 5.4E+3 | 6.2E+3 | 1.5E+4 | 1.3E+4 | 2.0E+4 | 1.4E+4 |
| HexP+4 | 1.1E+3 | 0.0E+0 | 2.3E+3 | 0.0E+0 | 7.6E+3 | 4.1E+3 | 9.3E+3 | 5.2E+3 | 5.1E+2 | 0.0E+0 | 0.0E+0 | 4.7E+2 | 7.2E+2 | 1.4E+3 | 8.9E+2 | 0.0E+0 |
|  |  |  |  |  |  |  |  |  |  |  |  |  |  |  |  |  |
| Asp | 1.6E+6 | 3.6E+6 | 9.6E+5 | 1.3E+6 | 1.2E+6 | 1.1E+6 | 1.1E+6 | 2.0E+6 | 3.6E+6 | 1.9E+6 | 8.1E+5 | 1.1E+6 | 1.3E+6 | 1.1E+6 | 4.4E+5 | 6.8E+5 |
| Asp+1 | 2.4E+4 | 6.3E+4 | 2.1E+5 | 4.5E+5 | 3.3E+5 | 4.6E+5 | 3.2E+5 | 5.3E+5 | 8.9E+4 | 3.2E+4 | 3.4E+5 | 3.3E+5 | 4.4E+5 | 4.0E+5 | 1.8E+5 | 2.3E+5 |
| Asp+2 | 1.6E+3 | 3.9E+3 | 8.8E+4 | 2.0E+5 | 1.9E+5 | 2.5E+5 | 2.1E+5 | 2.8E+5 | 1.3E+3 | 6.4E+2 | 1.4E+5 | 1.6E+5 | 2.2E+5 | 2.1E+5 | 7.5E+4 | 1.1E+5 |
| Asp+3 | 3.7E+3 | 4.1E+3 | 2.6E+4 | 6.0E+4 | 7.1E+4 | 7.7E+4 | 7.8E+4 | 6.5E+4 | 1.7E+4 | 3.6E+3 | 1.6E+4 | 3.2E+4 | 4.9E+4 | 5.4E+4 | 6.1E+3 | 2.7E+4 |
| Asp+4 | 1.1E+3 | 7.3E+2 | 1.8E+3 | 4.7E+3 | 5.4E+3 | 4.2E+3 | 4.9E+3 | 4.0E+3 | 5.2E+2 | 4.1E+2 | 2.3E+3 | 1.4E+3 | 3.0E+3 | 4.1E+3 | 0.0E+0 | 2.4E+3 |
|  |  |  |  |  |  |  |  |  |  |  |  |  |  |  |  |  |

| Table S2 Continued | | | |  | | |  | | | |  | | |  | | |  | | |  |
| --- | --- | --- | --- | --- | --- | --- | --- | --- | --- | --- | --- | --- | --- | --- | --- | --- | --- | --- | --- | --- |
|  | **Nitrogen Limited** | | | | | | | | | | | | | | | | | | | |
| metabolite isotopologue | 1200 | | | | | | | | | 1600 | | | | | | | | | | |
|  | 0 | | 30 | | 60 | | | 90 | | 0 | | | 30 | | | 60 | | | 90 | |
| S7P | 3.7E+6 | 4.7E+6 | 1.8E+6 | 1.6E+6 | 2.1E+6 | 2.1E+6 | | 1.4E+6 | 2.3E+6 | 4.3E+6 | | 2.5E+6 | 1.7E+6 | | 1.3E+6 | 1.2E+6 | | 1.3E+6 | 1.1E+6 | 9.3E+5 |
| S7P+1 | 1.2E+5 | 1.9E+5 | 7.2E+5 | 5.6E+5 | 1.0E+6 | 9.8E+5 | | 7.6E+5 | 9.4E+5 | 2.1E+5 | | 9.0E+4 | 6.1E+5 | | 4.2E+5 | 5.3E+5 | | 5.5E+5 | 5.4E+5 | 3.3E+5 |
| S7P+2 | 4.4E+3 | 2.3E+4 | 3.0E+5 | 1.8E+5 | 5.3E+5 | 3.8E+5 | | 4.6E+5 | 4.6E+5 | 2.0E+4 | | 7.9E+3 | 1.8E+5 | | 1.1E+5 | 2.0E+5 | | 1.8E+5 | 1.8E+5 | 1.4E+5 |
| S7P+3 | 0.0E+0 | 3.8E+3 | 6.9E+4 | 2.4E+4 | 2.0E+5 | 1.2E+5 | | 2.2E+5 | 1.9E+5 | 0.0E+0 | | 6.1E+2 | 3.5E+4 | | 2.4E+4 | 4.4E+4 | | 4.4E+4 | 3.0E+4 | 3.2E+4 |
| S7P+4 | 0.0E+0 | 0.0E+0 | 5.1E+3 | 5.7E+3 | 4.3E+4 | 1.1E+4 | | 5.0E+4 | 4.5E+4 | 0.0E+0 | | 0.0E+0 | 3.3E+3 | | 1.7E+3 | 3.4E+3 | | 5.2E+3 | 8.9E+2 | 1.8E+3 |
| S7P+5 | 0.0E+0 | 0.0E+0 | 1.6E+3 | 0.0E+0 | 7.0E+3 | 0.0E+0 | | 7.1E+3 | 2.8E+3 | 0.0E+0 | | 0.0E+0 | 0.0E+0 | | 0.0E+0 | 0.0E+0 | | 0.0E+0 | 1.1E+3 | 0.0E+0 |
| UDP-Glc | 7.0E+6 | 1.1E+7 | 4.3E+6 | 5.9E+6 | 4.5E+6 | 4.9E+6 | | 4.0E+6 | 4.8E+6 | 9.9E+6 | | 8.4E+6 | 4.6E+6 | | 5.3E+6 | 3.7E+6 | | 4.7E+6 | 2.0E+6 | 6.7E+6 |
| UDP-Glc+1 | 9.7E+5 | 1.6E+6 | 1.3E+6 | 1.9E+6 | 1.7E+6 | 2.0E+6 | | 1.9E+6 | 2.3E+6 | 1.5E+6 | | 1.3E+6 | 1.4E+6 | | 1.6E+6 | 1.4E+6 | | 1.9E+6 | 8.1E+5 | 2.1E+6 |
| UDP-Glc+2 | 9.6E+4 | 2.3E+5 | 7.8E+5 | 1.1E+6 | 1.4E+6 | 1.4E+6 | | 1.7E+6 | 1.9E+6 | 2.1E+5 | | 2.0E+5 | 7.2E+5 | | 8.0E+5 | 9.8E+5 | | 1.3E+6 | 6.0E+5 | 1.2E+6 |
| UDP-Glc+3 | 3.2E+3 | 0.0E+0 | 5.2E+5 | 6.1E+5 | 9.9E+5 | 9.4E+5 | | 1.3E+6 | 1.3E+6 | 1.7E+3 | | 0.0E+0 | 3.9E+5 | | 3.9E+5 | 5.8E+5 | | 7.8E+5 | 3.3E+5 | 7.2E+5 |
| UDP-Glc+4 | 0.0E+0 | 1.5E+3 | 2.8E+5 | 2.1E+5 | 5.6E+5 | 4.0E+5 | | 7.9E+5 | 6.9E+5 | 0.0E+0 | | 1.8E+3 | 1.4E+5 | | 1.5E+5 | 2.5E+5 | | 3.3E+5 | 1.6E+5 | 3.5E+5 |
| UDP-Glc+5 | 0.0E+0 | 5.2E+3 | 5.5E+4 | 5.0E+4 | 1.5E+5 | 9.6E+4 | | 2.6E+5 | 2.5E+5 | 0.0E+0 | | 0.0E+0 | 2.4E+4 | | 2.3E+4 | 7.4E+4 | | 9.3E+4 | 1.9E+4 | 6.1E+4 |
| UDP-Glc+6 | 0.0E+0 | 1.9E+3 | 8.2E+3 | 1.9E+3 | 9.2E+3 | 4.9E+3 | | 3.4E+4 | 3.4E+4 | 1.2E+3 | | 0.0E+0 | 5.5E+3 | | 0.0E+0 | 8.3E+3 | | 1.3E+4 | 2.0E+3 | 8.0E+3 |
| UDP-Glc+7 | 0.0E+0 | 0.0E+0 | 1.4E+3 | 1.7E+3 | 0.0E+0 | 5.8E+3 | | 7.7E+3 | 5.4E+3 | 0.0E+0 | | 0.0E+0 | 0.0E+0 | | 0.0E+0 | 0.0E+0 | | 1.2E+3 | 8.8E+3 | 0.0E+0 |
| Inosine | 9.4E+7 | 1.9E+8 | 9.2E+7 | 9.6E+7 | 1.1E+8 | 1.1E+8 | | 1.2E+8 | 1.4E+8 | 1.5E+8 | | 1.1E+8 | 6.5E+7 | | 6.8E+7 | 8.1E+7 | | 7.5E+7 | 6.4E+7 | 5.5E+7 |
| Inosine+1 | 8.5E+6 | 1.7E+7 | 9.5E+6 | 9.9E+6 | 1.2E+7 | 1.2E+7 | | 1.4E+7 | 1.6E+7 | 1.3E+7 | | 9.8E+6 | 6.7E+6 | | 7.0E+6 | 9.1E+6 | | 8.6E+6 | 8.0E+6 | 6.7E+6 |
| Inosine+2 | 1.2E+6 | 2.4E+6 | 9.6E+5 | 1.0E+6 | 1.5E+6 | 1.4E+6 | | 2.4E+6 | 2.6E+6 | 2.0E+6 | | 1.4E+6 | 6.5E+5 | | 7.0E+5 | 1.2E+6 | | 1.2E+6 | 1.5E+6 | 1.3E+6 |
| Inosine+3 | 5.6E+4 | 1.6E+5 | 5.4E+5 | 4.1E+5 | 1.3E+6 | 1.1E+6 | | 2.3E+6 | 2.3E+6 | 1.1E+5 | | 9.3E+4 | 2.9E+5 | | 2.9E+5 | 9.6E+5 | | 9.4E+5 | 1.2E+6 | 1.0E+6 |
| Inosine+4 | 0.0E+0 | 4.3E+3 | 4.0E+5 | 2.5E+5 | 1.1E+6 | 7.9E+5 | | 1.9E+6 | 1.8E+6 | 2.0E+3 | | 0.0E+0 | 1.9E+5 | | 1.9E+5 | 6.7E+5 | | 6.5E+5 | 8.7E+5 | 6.9E+5 |
| Inosine+5 | 0.0E+0 | 0.0E+0 | 2.3E+5 | 1.2E+5 | 6.6E+5 | 4.4E+5 | | 1.2E+6 | 9.9E+5 | 1.5E+3 | | 0.0E+0 | 6.5E+4 | | 7.6E+4 | 3.6E+5 | | 3.5E+5 | 4.8E+5 | 3.6E+5 |
| Inosine+6 | 2.3E+3 | 2.7E+3 | 9.4E+4 | 2.9E+4 | 3.2E+5 | 1.4E+5 | | 5.2E+5 | 3.8E+5 | 3.0E+3 | | 4.5E+3 | 1.8E+4 | | 1.8E+4 | 1.2E+5 | | 1.2E+5 | 1.6E+5 | 1.3E+5 |
| Inosine+7 | 0.0E+0 | 0.0E+0 | 3.5E+3 | 8.8E+3 | 7.3E+4 | 2.1E+4 | | 1.4E+5 | 9.2E+4 | 1.8E+3 | | 0.0E+0 | 2.4E+3 | | 1.1E+3 | 1.4E+4 | | 2.3E+4 | 2.6E+4 | 1.9E+4 |
|  |  |  |  |  |  |  | |  |  |  | |  |  | |  |  | |  |  |  |

| Table S2 Continued | | | |  | |  | |  | | |  | | |  | | |  | |  |
| --- | --- | --- | --- | --- | --- | --- | --- | --- | --- | --- | --- | --- | --- | --- | --- | --- | --- | --- | --- |
|  | **Nitrogen Limited** | | | | | | | | | | | | | | | | | | |
| metabolite isotopologue | 1200 | | | | | | | | | 1600 | | | | | | | | | |
|  | 0 | | 30 | | | 60 | | 90 | | 0 | | | 30 | | | 60 | | 90 | |
| UDP-GlcN | 2.2E+6 | 4.1E+6 | 1.0E+6 | | 2.4E+6 | 1.3E+6 | 1.5E+6 | 1.2E+6 | 1.0E+6 | 4.0E+6 | | 2.7E+6 | 1.4E+6 | | 1.5E+6 | 1.1E+6 | 1.4E+6 | 1.4E+5 | 1.2E+7 |
| UDP-GlcN+1 | 2.6E+5 | 6.4E+5 | 2.2E+5 | | 5.9E+5 | 2.6E+5 | 3.4E+5 | 2.3E+5 | 2.7E+5 | 6.1E+5 | | 4.1E+5 | 3.6E+5 | | 3.6E+5 | 2.3E+5 | 4.4E+5 | 4.5E+3 | 2.3E+6 |
| UDP-GlcN+2 | 1.5E+4 | 3.9E+4 | 6.0E+4 | | 1.7E+5 | 1.1E+5 | 1.1E+5 | 1.1E+5 | 2.1E+5 | 5.8E+4 | | 2.7E+4 | 1.3E+5 | | 1.2E+5 | 1.1E+5 | 1.7E+5 | 0.0E+0 | 6.1E+5 |
| UDP-GlcN+3 | 1.5E+4 | 3.9E+4 | 6.0E+4 | | 1.8E+5 | 1.1E+5 | 1.1E+5 | 1.1E+5 | 2.1E+5 | 6.0E+4 | | 2.7E+4 | 1.3E+5 | | 1.2E+5 | 1.1E+5 | 1.7E+5 | 0.0E+0 | 6.1E+5 |
| UDP-GlcN+4 | 0.0E+0 | 0.0E+0 | 3.1E+4 | | 5.3E+4 | 6.4E+4 | 6.1E+4 | 1.1E+5 | 1.1E+5 | 0.0E+0 | | 0.0E+0 | 3.7E+4 | | 4.6E+4 | 4.3E+4 | 7.0E+4 | 9.5E+3 | 1.3E+5 |
| UDP-GlcN+5 | 0.0E+0 | 3.9E+3 | 6.4E+3 | | 2.3E+4 | 3.6E+4 | 3.4E+4 | 6.4E+4 | 5.6E+4 | 0.0E+0 | | 0.0E+0 | 1.1E+4 | | 1.4E+4 | 2.6E+4 | 4.9E+4 | 0.0E+0 | 4.6E+4 |
| UDP-GlcN+6 | 8.5E+3 | 1.6E+3 | 6.6E+3 | | 0.0E+0 | 1.1E+4 | 6.0E+3 | 2.0E+4 | 2.1E+4 | 0.0E+0 | | 0.0E+0 | 3.3E+3 | | 0.0E+0 | 8.5E+3 | 1.3E+4 | 0.0E+0 | 1.7E+4 |
| UDP-GlcN+7 | 0.0E+0 | 0.0E+0 | 1.4E+3 | | 2.2E+3 | 3.6E+3 | 0.0E+0 | 1.6E+3 | 0.0E+0 | 0.0E+0 | | 0.0E+0 | 0.0E+0 | | 4.9E+3 | 0.0E+0 | 1.1E+3 | 0.0E+0 | 1.6E+3 |
| UDP-GlcN+8 | 0.0E+0 | 0.0E+0 | 0.0E+0 | | 6.7E+3 | 2.1E+3 | 0.0E+0 | 1.5E+3 | 0.0E+0 | 0.0E+0 | | 1.4E+3 | 0.0E+0 | | 1.6E+3 | 0.0E+0 | 0.0E+0 | 0.0E+0 | 0.0E+0 |
|  |  |  |  | |  |  |  |  |  |  | |  |  | |  |  |  |  |  |
|  | **Nitrogen Replete** | | | | | | | | | | | | | | | | | | |
| metabolite isotopologue | 1200 | | | | | | | | | 1600 | | | | | | | | | |
|  | 0 | | 30 | | | 60 | | 90 | | 0 | | | 30 | | | 60 | | 90 | |
| Glu | 2.2E+8 | 2.3E+8 | 1.9E+8 | | 2.2E+8 | 1.9E+8 | 1.9E+8 | 2.1E+8 | 2.5E+8 | 2.8E+8 | | 1.8E+8 | 2.2E+8 | | 2.1E+8 | 2.3E+8 | 2.0E+8 | 2.4E+8 | 2.5E+8 |
| Glu +1 | 8.6E+6 | 8.7E+6 | 8.2E+6 | | 1.1E+7 | 1.1E+7 | 1.0E+7 | 1.4E+7 | 1.6E+7 | 1.1E+7 | | 6.7E+6 | 9.3E+6 | | 1.1E+7 | 1.4E+7 | 1.2E+7 | 1.5E+7 | 1.8E+7 |
| Glu +2 | 5.6E+5 | 6.1E+5 | 7.0E+5 | | 7.8E+5 | 1.5E+6 | 1.6E+6 | 3.2E+6 | 3.7E+6 | 7.3E+5 | | 5.9E+5 | 7.4E+5 | | 7.6E+5 | 1.6E+6 | 1.7E+6 | 2.8E+6 | 4.2E+6 |
| Glu +3 | 1.5E+4 | 2.9E+4 | 2.6E+5 | | 2.9E+5 | 6.3E+5 | 6.8E+5 | 1.5E+6 | 1.7E+6 | 3.1E+4 | | 8.8E+3 | 2.4E+5 | | 3.1E+5 | 7.2E+5 | 7.1E+5 | 1.2E+6 | 1.8E+6 |
| Glu +4 | 3.9E+4 | 3.6E+4 | 7.3E+4 | | 1.1E+5 | 2.5E+5 | 2.4E+5 | 5.3E+5 | 6.0E+5 | 3.1E+4 | | 1.1E+3 | 6.6E+4 | | 1.0E+5 | 2.5E+5 | 2.2E+5 | 3.6E+5 | 5.5E+5 |
| HexP | 3.3E+6 | 3.6E+6 | 6.9E+5 | | 7.0E+5 | 5.0E+5 | 5.5E+5 | 4.2E+5 | 5.6E+5 | 4.3E+6 | | 3.8E+6 | 1.2E+6 | | 1.2E+6 | 1.0E+6 | 9.2E+5 | 1.1E+6 | 1.3E+6 |
| HexP+1 | 1.1E+5 | 8.8E+4 | 3.7E+5 | | 4.0E+5 | 3.6E+5 | 3.5E+5 | 4.0E+5 | 4.0E+5 | 1.2E+5 | | 1.3E+5 | 6.4E+5 | | 7.3E+5 | 6.4E+5 | 6.3E+5 | 6.9E+5 | 8.4E+5 |
| HexP+2 | 4.3E+3 | 1.9E+4 | 5.3E+5 | | 5.8E+5 | 5.7E+5 | 6.3E+5 | 6.7E+5 | 8.5E+5 | 1.2E+4 | | 1.7E+3 | 5.5E+5 | | 6.5E+5 | 5.8E+5 | 5.6E+5 | 5.2E+5 | 6.7E+5 |
| HexP+3 | 0.0E+0 | 1.2E+3 | 2.3E+5 | | 2.8E+5 | 2.7E+5 | 2.3E+5 | 3.7E+5 | 3.9E+5 | 1.7E+3 | | 4.6E+2 | 2.1E+5 | | 2.7E+5 | 2.2E+5 | 2.2E+5 | 1.9E+5 | 2.5E+5 |
| HexP+4 | 1.5E+3 | 0.0E+0 | 3.5E+4 | | 3.9E+4 | 3.3E+4 | 3.6E+4 | 8.3E+4 | 6.4E+4 | 0.0E+0 | | 0.0E+0 | 2.4E+4 | | 4.2E+4 | 3.0E+4 | 2.6E+4 | 1.7E+4 | 3.1E+4 |

| Table S2 Continued | |  |  |  |  |  |  |  |  |  | |  | |  |  |  |  |  |
| --- | --- | --- | --- | --- | --- | --- | --- | --- | --- | --- | --- | --- | --- | --- | --- | --- | --- | --- |
|  | **Nitrogen Replete** | | | | | | | | | | | | | | | | |  |
| metabolite isotopologue | 1200 | | | | | | | | 1600 | | | | | | | | |  |
|  | 0 | | 30 | | 60 | | 90 | | 0 | | 30 | | | 60 | | 90 | |  |
| Asp | 5.8E+6 | 6.6E+6 | 3.2E+6 | 4.3E+6 | 2.5E+6 | 3.0E+6 | 2.8E+6 | 3.1E+6 | 8.4E+6 | 5.8E+6 | 4.8E+6 | | 5.8E+6 | 4.6E+6 | 4.3E+6 | 3.9E+6 | 5.6E+6 |  |
| Asp+1 | 1.1E+5 | 1.3E+5 | 2.6E+5 | 2.9E+5 | 3.3E+5 | 3.7E+5 | 5.2E+5 | 6.1E+5 | 1.5E+5 | 1.0E+5 | 3.1E+5 | | 3.6E+5 | 4.8E+5 | 4.4E+5 | 6.3E+5 | 8.3E+5 |  |
| Asp+2 | 7.1E+2 | 1.6E+3 | 1.7E+5 | 2.1E+5 | 3.3E+5 | 3.9E+5 | 6.3E+5 | 7.0E+5 | 5.2E+3 | 3.6E+3 | 2.0E+5 | | 2.6E+5 | 4.3E+5 | 4.4E+5 | 6.1E+5 | 8.5E+5 |  |
| Asp+3 | 3.3E+3 | 1.3E+4 | 9.4E+4 | 1.2E+5 | 2.2E+5 | 2.1E+5 | 3.9E+5 | 3.9E+5 | 1.1E+4 | 2.3E+3 | 8.6E+4 | | 1.1E+5 | 2.0E+5 | 2.1E+5 | 3.3E+5 | 4.3E+5 |  |
| Asp+4 | 5.2E+2 | 6.0E+2 | 4.1E+3 | 1.2E+4 | 1.4E+4 | 2.6E+4 | 6.5E+4 | 6.9E+4 | 7.1E+2 | 0.0E+0 | 9.6E+3 | | 7.3E+3 | 2.6E+4 | 2.8E+4 | 3.8E+4 | 6.6E+4 |  |
| S7P | 1.1E+6 | 1.5E+6 | 1.2E+5 | 1.0E+5 | 4.3E+4 | 7.3E+4 | 8.3E+4 | 7.9E+4 | 1.3E+6 | 1.2E+6 | 2.3E+5 | | 2.1E+5 | 1.8E+5 | 1.1E+5 | 2.4E+5 | 2.1E+5 |  |
| S7P+1 | 3.1E+4 | 2.6E+4 | 9.5E+4 | 9.7E+4 | 3.3E+4 | 4.0E+4 | 5.0E+4 | 5.4E+4 | 2.3E+4 | 2.1E+4 | 1.7E+5 | | 1.7E+5 | 1.3E+5 | 1.1E+5 | 1.7E+5 | 1.5E+5 |  |
| S7P+2 | 3.8E+3 | 8.9E+3 | 1.8E+5 | 1.8E+5 | 9.1E+4 | 1.0E+5 | 1.3E+5 | 1.5E+5 | 4.1E+3 | 0.0E+0 | 1.9E+5 | | 2.1E+5 | 1.6E+5 | 1.5E+5 | 1.6E+5 | 2.3E+5 |  |
| S7P+3 | 2.1E+3 | 0.0E+0 | 1.9E+5 | 1.9E+5 | 1.2E+5 | 1.2E+5 | 1.4E+5 | 2.1E+5 | 0.0E+0 | 0.0E+0 | 1.5E+5 | | 1.8E+5 | 1.7E+5 | 1.2E+5 | 1.1E+5 | 1.6E+5 |  |
| S7P+4 | 0.0E+0 | 0.0E+0 | 1.3E+5 | 1.1E+5 | 7.7E+4 | 8.1E+4 | 1.1E+5 | 1.4E+5 | 0.0E+0 | 5.5E+2 | 7.9E+4 | | 1.0E+5 | 5.8E+4 | 5.1E+4 | 3.4E+4 | 5.8E+4 |  |
| S7P+5 | 0.0E+0 | 0.0E+0 | 3.4E+4 | 3.6E+4 | 9.2E+3 | 7.0E+3 | 3.7E+4 | 3.1E+4 | 0.0E+0 | 0.0E+0 | 1.2E+4 | | 2.5E+4 | 1.7E+4 | 7.2E+3 | 5.2E+3 | 1.2E+4 |  |
| UDP-Glc | 3.7E+6 | 5.8E+6 | 5.0E+5 | 5.0E+5 | 1.4E+5 | 1.8E+5 | 1.3E+5 | 3.3E+5 | 3.7E+6 | 3.5E+6 | 6.5E+5 | | 5.9E+5 | 3.0E+5 | 2.8E+5 | 1.7E+5 | 4.7E+5 |  |
| UDP-Glc+1 | 5.1E+5 | 7.8E+5 | 4.1E+5 | 4.2E+5 | 2.3E+5 | 2.8E+5 | 2.8E+5 | 2.8E+5 | 5.3E+5 | 4.2E+5 | 5.0E+5 | | 4.5E+5 | 3.8E+5 | 2.9E+5 | 2.2E+5 | 4.0E+5 |  |
| UDP-Glc+2 | 4.1E+4 | 4.8E+4 | 7.9E+5 | 8.2E+5 | 6.9E+5 | 6.5E+5 | 6.6E+5 | 7.2E+5 | 4.9E+4 | 3.7E+4 | 7.0E+5 | | 6.7E+5 | 6.3E+5 | 7.1E+5 | 5.2E+5 | 7.0E+5 |  |
| UDP-Glc+3 | 1.5E+3 | 0.0E+0 | 8.9E+5 | 9.5E+5 | 8.9E+5 | 7.7E+5 | 8.0E+5 | 8.7E+5 | 2.3E+3 | 1.8E+3 | 7.3E+5 | | 6.9E+5 | 7.4E+5 | 7.7E+5 | 5.5E+5 | 8.1E+5 |  |
| UDP-Glc+4 | 0.0E+0 | 0.0E+0 | 5.5E+5 | 6.2E+5 | 5.6E+5 | 5.5E+5 | 6.2E+5 | 7.3E+5 | 0.0E+0 | 0.0E+0 | 5.0E+5 | | 4.6E+5 | 5.2E+5 | 6.0E+5 | 3.8E+5 | 5.5E+5 |  |
| UDP-Glc+5 | 0.0E+0 | 0.0E+0 | 2.6E+5 | 2.2E+5 | 1.5E+5 | 2.7E+5 | 3.1E+5 | 3.2E+5 | 0.0E+0 | 0.0E+0 | 2.2E+5 | | 1.9E+5 | 2.4E+5 | 2.9E+5 | 2.2E+5 | 3.1E+5 |  |
| UDP-Glc+6 | 1.2E+3 | 0.0E+0 | 3.1E+4 | 3.6E+4 | 4.3E+4 | 1.0E+5 | 1.4E+5 | 1.9E+5 | 2.4E+3 | 0.0E+0 | 5.5E+4 | | 5.3E+4 | 9.5E+4 | 1.1E+5 | 1.2E+5 | 1.9E+5 |  |
| UDP-Glc+7 | 0.0E+0 | 0.0E+0 | 6.8E+3 | 4.6E+3 | 1.9E+4 | 3.0E+4 | 8.1E+4 | 6.7E+4 | 0.0E+0 | 0.0E+0 | 1.2E+4 | | 1.3E+4 | 4.2E+4 | 5.8E+4 | 6.3E+4 | 8.7E+4 |  |
|  |  |  |  |  |  |  |  |  |  |  |  | |  |  |  |  |  |  |
|  |  |  |  |  |  |  |  |  |  |  |  | |  |  |  |  |  |  |
|  |  |  |  |  |  |  |  |  |  |  |  | |  |  |  |  |  |  |
|  |  |  |  |  |  |  |  |  |  |  |  | |  |  |  |  |  |  |

| Table S2 Continued | |  |  |  |  |  |  |  |  |  | |  | |  |  |  |  |  |
| --- | --- | --- | --- | --- | --- | --- | --- | --- | --- | --- | --- | --- | --- | --- | --- | --- | --- | --- |
|  | **Nitrogen Replete** | | | | | | | | | | | | | | | | |  |
| metabolite isotopologue | 1200 | | | | | | | | 1600 | | | | | | | | |  |
|  | 0 | | 30 | | 60 | | 90 | | 0 | | 30 | | | 60 | | 90 | |  |
| Asp | 5.8E+6 | 6.6E+6 | 3.2E+6 | 4.3E+6 | 2.5E+6 | 3.0E+6 | 2.8E+6 | 3.1E+6 | 8.4E+6 | 5.8E+6 | 4.8E+6 | | 5.8E+6 | 4.6E+6 | 4.3E+6 | 3.9E+6 | 5.6E+6 |  |
| Asp+1 | 1.1E+5 | 1.3E+5 | 2.6E+5 | 2.9E+5 | 3.3E+5 | 3.7E+5 | 5.2E+5 | 6.1E+5 | 1.5E+5 | 1.0E+5 | 3.1E+5 | | 3.6E+5 | 4.8E+5 | 4.4E+5 | 6.3E+5 | 8.3E+5 |  |
| Asp+2 | 7.1E+2 | 1.6E+3 | 1.7E+5 | 2.1E+5 | 3.3E+5 | 3.9E+5 | 6.3E+5 | 7.0E+5 | 5.2E+3 | 3.6E+3 | 2.0E+5 | | 2.6E+5 | 4.3E+5 | 4.4E+5 | 6.1E+5 | 8.5E+5 |  |
| Asp+3 | 3.3E+3 | 1.3E+4 | 9.4E+4 | 1.2E+5 | 2.2E+5 | 2.1E+5 | 3.9E+5 | 3.9E+5 | 1.1E+4 | 2.3E+3 | 8.6E+4 | | 1.1E+5 | 2.0E+5 | 2.1E+5 | 3.3E+5 | 4.3E+5 |  |
| Asp+4 | 5.2E+2 | 6.0E+2 | 4.1E+3 | 1.2E+4 | 1.4E+4 | 2.6E+4 | 6.5E+4 | 6.9E+4 | 7.1E+2 | 0.0E+0 | 9.6E+3 | | 7.3E+3 | 2.6E+4 | 2.8E+4 | 3.8E+4 | 6.6E+4 |  |
| S7P | 1.1E+6 | 1.5E+6 | 1.2E+5 | 1.0E+5 | 4.3E+4 | 7.3E+4 | 8.3E+4 | 7.9E+4 | 1.3E+6 | 1.2E+6 | 2.3E+5 | | 2.1E+5 | 1.8E+5 | 1.1E+5 | 2.4E+5 | 2.1E+5 |  |
| S7P+1 | 3.1E+4 | 2.6E+4 | 9.5E+4 | 9.7E+4 | 3.3E+4 | 4.0E+4 | 5.0E+4 | 5.4E+4 | 2.3E+4 | 2.1E+4 | 1.7E+5 | | 1.7E+5 | 1.3E+5 | 1.1E+5 | 1.7E+5 | 1.5E+5 |  |
| S7P+2 | 3.8E+3 | 8.9E+3 | 1.8E+5 | 1.8E+5 | 9.1E+4 | 1.0E+5 | 1.3E+5 | 1.5E+5 | 4.1E+3 | 0.0E+0 | 1.9E+5 | | 2.1E+5 | 1.6E+5 | 1.5E+5 | 1.6E+5 | 2.3E+5 |  |
| S7P+3 | 2.1E+3 | 0.0E+0 | 1.9E+5 | 1.9E+5 | 1.2E+5 | 1.2E+5 | 1.4E+5 | 2.1E+5 | 0.0E+0 | 0.0E+0 | 1.5E+5 | | 1.8E+5 | 1.7E+5 | 1.2E+5 | 1.1E+5 | 1.6E+5 |  |
| S7P+4 | 0.0E+0 | 0.0E+0 | 1.3E+5 | 1.1E+5 | 7.7E+4 | 8.1E+4 | 1.1E+5 | 1.4E+5 | 0.0E+0 | 5.5E+2 | 7.9E+4 | | 1.0E+5 | 5.8E+4 | 5.1E+4 | 3.4E+4 | 5.8E+4 |  |
| S7P+5 | 0.0E+0 | 0.0E+0 | 3.4E+4 | 3.6E+4 | 9.2E+3 | 7.0E+3 | 3.7E+4 | 3.1E+4 | 0.0E+0 | 0.0E+0 | 1.2E+4 | | 2.5E+4 | 1.7E+4 | 7.2E+3 | 5.2E+3 | 1.2E+4 |  |
| UDP-Glc | 3.7E+6 | 5.8E+6 | 5.0E+5 | 5.0E+5 | 1.4E+5 | 1.8E+5 | 1.3E+5 | 3.3E+5 | 3.7E+6 | 3.5E+6 | 6.5E+5 | | 5.9E+5 | 3.0E+5 | 2.8E+5 | 1.7E+5 | 4.7E+5 |  |
| UDP-Glc+1 | 5.1E+5 | 7.8E+5 | 4.1E+5 | 4.2E+5 | 2.3E+5 | 2.8E+5 | 2.8E+5 | 2.8E+5 | 5.3E+5 | 4.2E+5 | 5.0E+5 | | 4.5E+5 | 3.8E+5 | 2.9E+5 | 2.2E+5 | 4.0E+5 |  |
| UDP-Glc+2 | 4.1E+4 | 4.8E+4 | 7.9E+5 | 8.2E+5 | 6.9E+5 | 6.5E+5 | 6.6E+5 | 7.2E+5 | 4.9E+4 | 3.7E+4 | 7.0E+5 | | 6.7E+5 | 6.3E+5 | 7.1E+5 | 5.2E+5 | 7.0E+5 |  |
| UDP-Glc+3 | 1.5E+3 | 0.0E+0 | 8.9E+5 | 9.5E+5 | 8.9E+5 | 7.7E+5 | 8.0E+5 | 8.7E+5 | 2.3E+3 | 1.8E+3 | 7.3E+5 | | 6.9E+5 | 7.4E+5 | 7.7E+5 | 5.5E+5 | 8.1E+5 |  |
| UDP-Glc+4 | 0.0E+0 | 0.0E+0 | 5.5E+5 | 6.2E+5 | 5.6E+5 | 5.5E+5 | 6.2E+5 | 7.3E+5 | 0.0E+0 | 0.0E+0 | 5.0E+5 | | 4.6E+5 | 5.2E+5 | 6.0E+5 | 3.8E+5 | 5.5E+5 |  |
| UDP-Glc+5 | 0.0E+0 | 0.0E+0 | 2.6E+5 | 2.2E+5 | 1.5E+5 | 2.7E+5 | 3.1E+5 | 3.2E+5 | 0.0E+0 | 0.0E+0 | 2.2E+5 | | 1.9E+5 | 2.4E+5 | 2.9E+5 | 2.2E+5 | 3.1E+5 |  |
| UDP-Glc+6 | 1.2E+3 | 0.0E+0 | 3.1E+4 | 3.6E+4 | 4.3E+4 | 1.0E+5 | 1.4E+5 | 1.9E+5 | 2.4E+3 | 0.0E+0 | 5.5E+4 | | 5.3E+4 | 9.5E+4 | 1.1E+5 | 1.2E+5 | 1.9E+5 |  |
| UDP-Glc+7 | 0.0E+0 | 0.0E+0 | 6.8E+3 | 4.6E+3 | 1.9E+4 | 3.0E+4 | 8.1E+4 | 6.7E+4 | 0.0E+0 | 0.0E+0 | 1.2E+4 | | 1.3E+4 | 4.2E+4 | 5.8E+4 | 6.3E+4 | 8.7E+4 |  |
|  |  |  |  |  |  |  |  |  |  |  |  | |  |  |  |  |  |  |
|  |  |  |  |  |  |  |  |  |  |  |  | |  |  |  |  |  |  |
|  |  |  |  |  |  |  |  |  |  |  |  | |  |  |  |  |  |  |
|  |  |  |  |  |  |  |  |  |  |  |  | |  |  |  |  |  |  |
