# Supplementary material for: Carbon Fate and Flux in Prochlorococcus under Nitrogen Limitation
Source: mSystems. 2019 Feb 26;4(1):e00254-18. doi: 10.1128/mSystems.00254-18 (PMC6392094; doi:10.1128/mSystems.00254-18)
Supplement: TABLE S3 [file mSystems.00254-18-st003.docx]

| Experiment | Date | Latitude | Longitude | Surface  Temp (°C) | Ammonium  concentration  (nM) | *Prochlorococcus* (cells ml^-1^) | *Synechococcus*  (cells ml^-1^) | Picoeukaryotes  (cells ml^-1^) |
| --- | --- | --- | --- | --- | --- | --- | --- | --- |
| Jan. Exp. 1 | 1/12/13 | 26° 41' N | 158° 0.0' W | 23 | 29 | 1.77E+05 | 1.59E+03 | 7.86E+02 |
| Jan. Exp. 2 | 1/14/13 | 33° 20' N | 159° 0.2' W | 18 | 7.7 | 1.19E+05 | 1.20E+03 | 1.51E+03 |
| Jan. Exp. 3 | 1/19/13 | 36° 32' N | 158° 40' W | 15 | 45.3 | 5.51E+04 | 7.44E+03 | 7.34E+03 |
| July Exp. 1 | 7/4/13 | 30° 43' N | 158° 0.0' W | 23 | 0 | 1.61E+05 | 1.09E+03 | 8.45E+02 |
| July Exp. 2 | 7/11/13 | 40° 61' N | 151° 47' W | 17 | 0 | 1.01E+05 | 9.62E+03 | 1.80E+03 |
| July Exp. 3 | 7/16/13 | 36° 88' N | 140° 60' W | 20 | 11.7 | 1.00E+05 | 4.90E+03 | 1.77E+03 |
