# Supplementary figures and images for: Carbon Fate and Flux in Prochlorococcus under Nitrogen Limitation
Source: mSystems. 2019 Feb 26;4(1):e00254-18. doi: 10.1128/mSystems.00254-18 (PMC6392094; doi:10.1128/mSystems.00254-18)

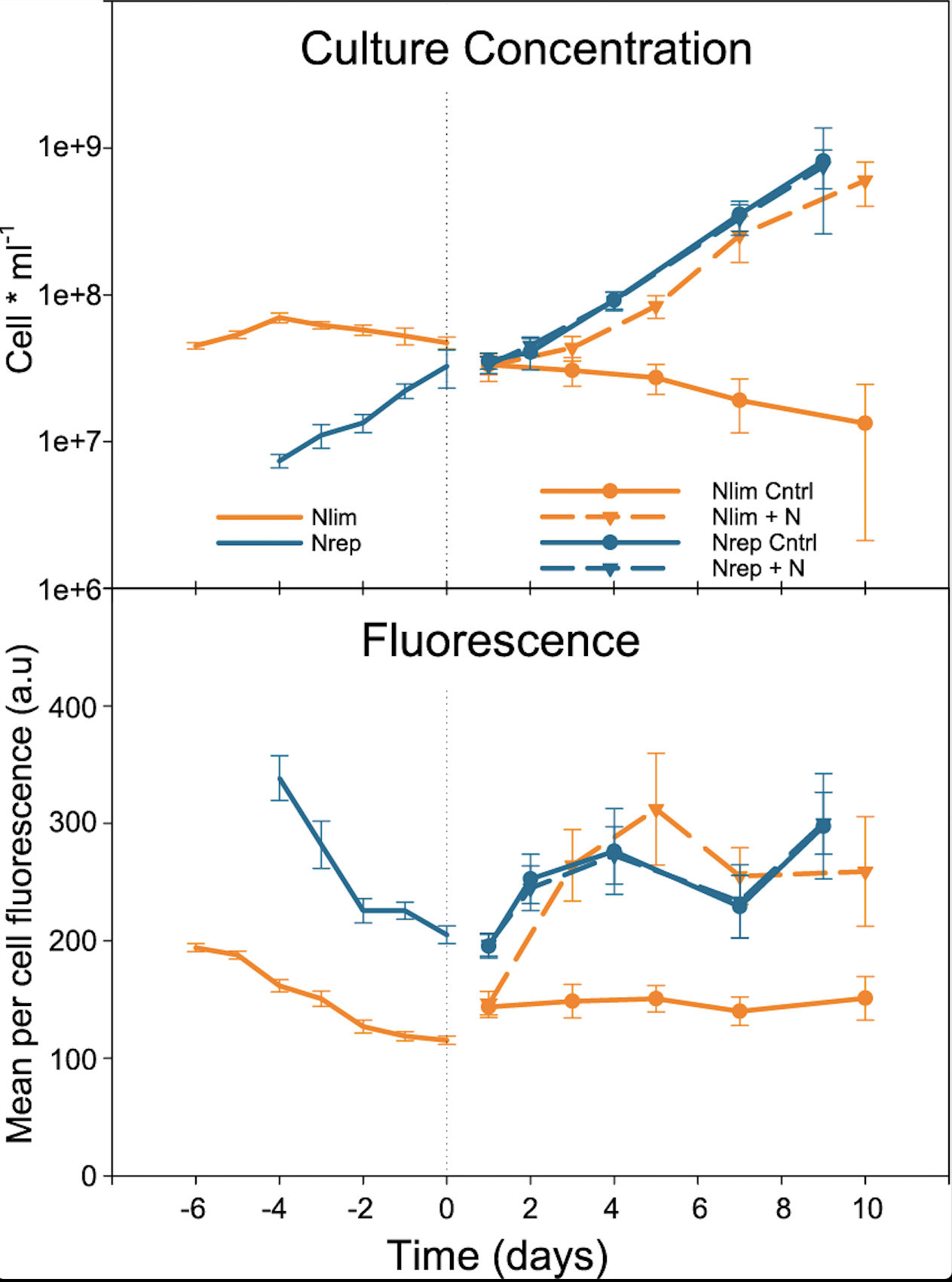

Supplement: FIG S1 [file mSystems.00254-18-sf001.jpg]

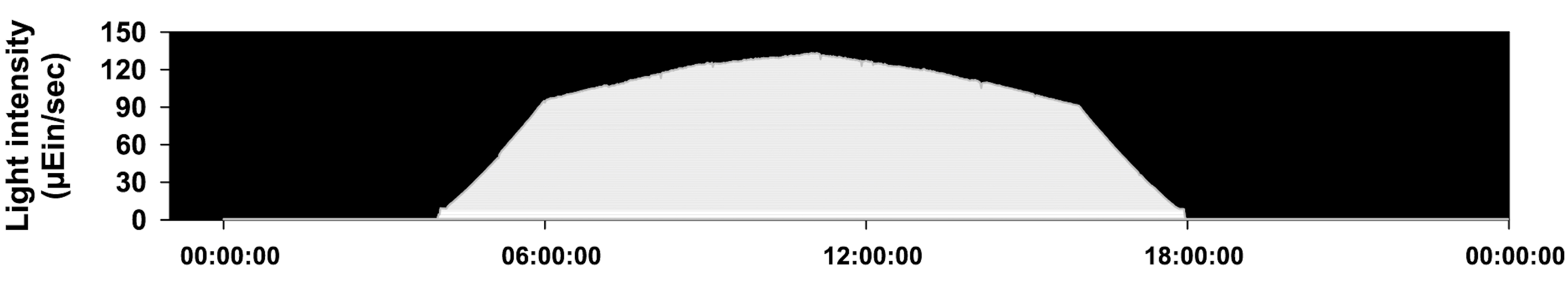

Supplement: FIG S2 [file mSystems.00254-18-sf002.jpg]

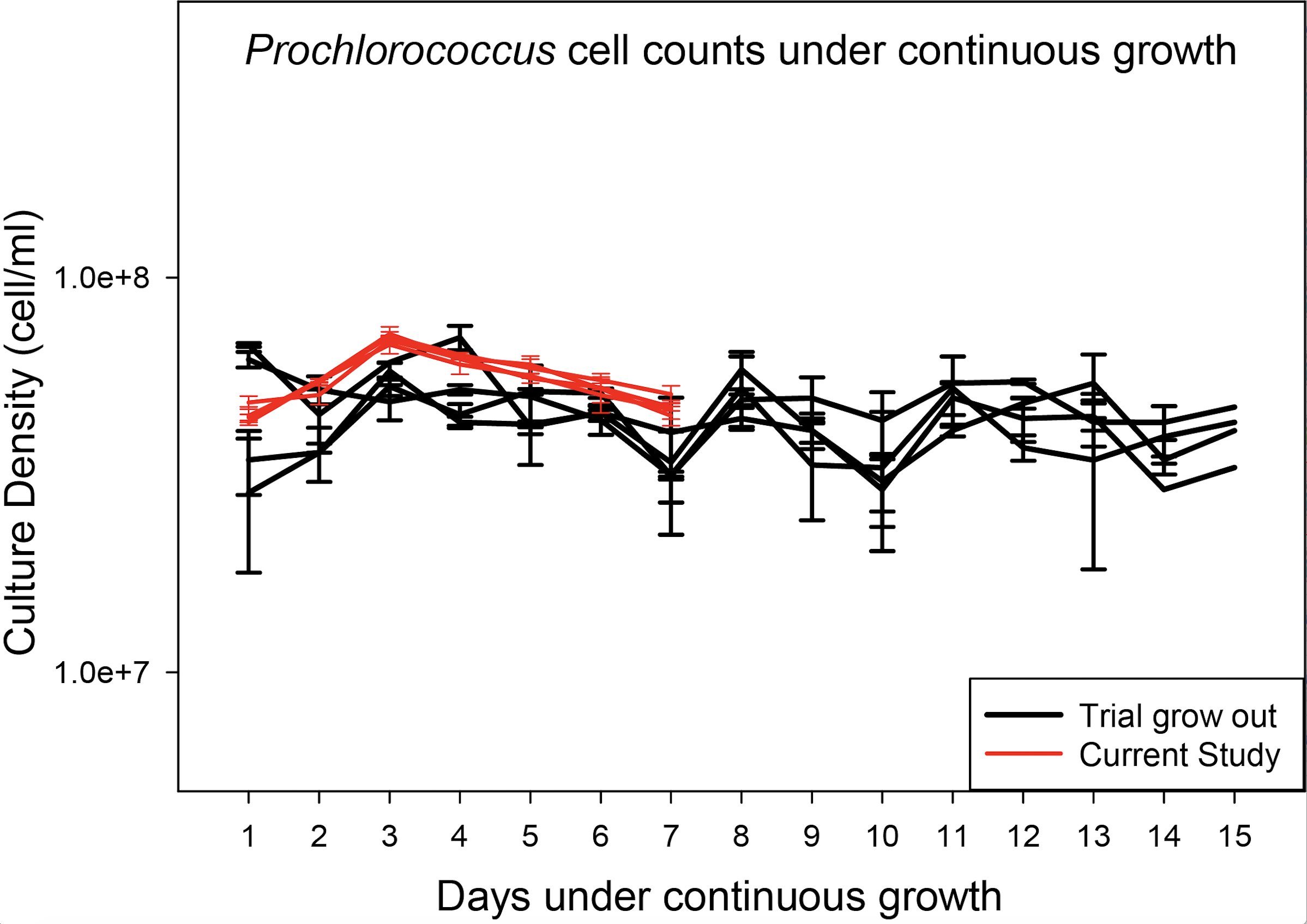

Supplement: FIG S3 [file mSystems.00254-18-sf003.jpg]
